# Endosomal Phosphatidylinositol-3-Phosphate-Associated Functions Are Dispensable for Establishment of the Cytomegalovirus Pre-Assembly Compartment but Essential for the Virus Growth

**DOI:** 10.3390/life11080859

**Published:** 2021-08-22

**Authors:** Marina Marcelić, Hana Mahmutefendić Lučin, Antonija Jurak Begonja, Gordana Blagojević Zagorac, Vanda Juranić Lisnić, Pero Lučin

**Affiliations:** 1 Department of Physiology and Immunology, Faculty of Medicine, University of Rijeka, 51000 Rijeka, Croatia; mmarcelic@uniri.hr (M.M.); hana.mahmutefendic@uniri.hr (H.M.L.); gordana.blagojevic@uniri.hr (G.B.Z.); 2 University North, University Center Varaždin, Jurja Križanića 31b, 42000 Varaždin, Croatia; 3 Department of Biotechnology, University of Rijeka, Radmile Matejčić 2, 51000 Rijeka, Croatia; ajbegonja@bioteh.uniri.hr; 4 Center for Proteomics, Department of Histology and Embryology, Faculty of Medicine, University of Rijeka, 51000 Rijeka, Croatia; vanda.juranic@uniri.hr

**Keywords:** cytomegalovirus, assembly compartment, early endosomes, endosomal recycling compartment, phosphatidylinositol-3-phosphate, Vps34, Rab10, Evectin-2, VPS34-IN1

## Abstract

Murine cytomegalovirus (MCMV) initiates the stepwise establishment of the pre-assembly compartment (pre-AC) in the early phase of infection by the expansion of the early endosome (EE)/endosomal recycling compartment (ERC) interface and relocation of the Golgi complex. We depleted Vps34-derived phosphatidylinositol-3-phosphate (PI(3)P) at EEs by VPS34-IN1 and inhibited PI(3)P-associated functions by overexpression of 2xFYVE- and p40PX PI(3)P-binding modules to assess the role of PI(3)P-dependent EE domains in the pre-AC biogenesis. We monitored the accumulation of Rab10 and Evectin-2 in the inner pre-AC and the relocation of GM130-positive cis-Golgi organelles to the outer pre-AC by confocal microscopy. Although PI(3)P- and Vps34-positive endosomes build a substantial part of pre-AC, the PI(3)P depletion and the inhibition of PI(3)P-associated functions did not prevent the establishment of infection and progression through the early phase. The PI(3)P depletion in uninfected and MCMV-infected cells rapidly dispersed PI(3)P-bond proteins and reorganized EEs, including ablation of EE-to-ERC transport and relocation of Rab11 endosomes. The PI(3)P depletion one hour before pre-AC initiation and overexpression of 2xFYVE and p40PX domains neither prevented Rab10- and Evectin-2 accumulation, nor Golgi unlinking and relocation. These data demonstrate that PI(3)P-dependent functions, including the Rab11-dependent EE-to-ERC route, are dispensable for pre-AC initiation. Nevertheless, the virus growth was drastically reduced in PI(3)P-depleted cells, indicating that PI(3)P-associated functions are essential for the late phase of infection.

## 1. Introduction

Cytomegaloviruses (CMV) are large DNA viruses that extensively reorganize infected cells. They arrange the membranous system of the cell into a large factory for the final packaging of nascent virions, known as the cytoplasmic assembly compartment (AC). This structure occupies an area as large as the nucleus and contains reorganized membranous organelles, which accumulate viral structural proteins required for the final packaging of nascent capsids into membrane-enveloped virions [1,2].

Studies on human CMV (HCMV) [3,4,5,6] and murine CMV (MCMV) [7,8] demonstrated that the development of the AC follows a similar set of events which involves reorganization of the Golgi and the endosomal system into the new organelle composition. More than three thousand host-cell genes may contribute to the organization of these organelles, and almost 1500 are significantly altered during HCMV infection [3]. The establishment of the AC occurs sequentially throughout the CMV replication cycle [8,9] and involves the interaction of a large number of viral proteins with normal host-cell functions [3,5]. Despite the significant progress in understanding the membranous organelle dynamics, these events are still insufficiently understood, as are host-cell functions targeted by CMVs. Given that the program of host-cell reorganization seems to be conserved among beta-herpesviruses, studies on HCMV and MCMV are supplementary and, therefore, might contribute to understanding the biology of AC development, as well as normal physiology of the host-cell membranous system.

In our recent study [8], we demonstrated that MCMV initiates the development of the AC very early in the infection. The basic structure, which is presented as a large perinuclear aggregate of membranous elements, is established in the early (E) phase of infection (at 4–6 hpi) and evolves throughout the E phase (up to 16 hpi). This structure can be considered as the pre-AC [8,10]. After initiating viral DNA synthesis at the beginning of the late phase of infection (15–16 hpi) and the expression of late genes, the aggregate is loaded by CMV-encoded glycoproteins and tegument proteins. At this stage, the structure becomes the AC, ready for acquiring nascent capsids and their final envelopment into newly formed virions. The virion egress occurs already at 20 hpi.

One of the earliest events identified in the pre-AC development is unlinking the Golgi ribbon and dislocation of the Golgi stacks associated with the expansion of membranous elements derived from early endosomes (EE), endosomal recycling compartment (ERC), and the trans-Golgi network (TGN) [8,11]. The EE-ERC-TGN interface involves intense membrane flow and cargo exchange between master compartments tightly compacted around the cell center, including many highly dynamic intermediates [12,13,14]. The homeostasis of the EE-ERC-TGN interface is balanced by a constant exchange of membranes between EEs and the TGN, incoming flux from EEs to the ERC, and outgoing flux from the ERC towards the PM, either directly or indirectly via the TGN [14]. Given that several regulatory proteins that characterize the ERC and the EE-ERC intermediates have been found recruited at membranes of the inner pre-AC [7,8,11], the dysregulation of membrane flow at this interface may be one of the initial steps in the biogenesis of the pre-AC.

The flux from EEs to the ERC and the biogenesis of the ERC remain unresolved [12]. A major driving force for maturation and sorting activities within EEs is the generation of the phosphatidylinositol-3-phosphate (PI(3)P) by the Rab5-recruited class III phosphatidylinositol-3-kinase (PI3K) complex containing Vps34 [15,16]. The localized PI(3)P production represents a platform for recruitment of effector proteins with specific PI(3)P-binding domains, such as the FYVE (Fab1, YOTB, Vac1, and EEA1 zinc-finger) and PX (Phox homology) domain [16] that drive cargo sorting, fusion, motility, and further maturation of EEs [17]. Most EE intermediates fuse and develop vacuolar endosomes that ultimately mature into LE, a complex PI(3)P-dependent process that involves Rab5-to-Rab7 conversion [16,18]. Along the maturation sequence of EEs, a substantial fraction of EE membranes is segregated into a domain that recruits Rab4 and generates tubules for cargo sorting and recycling back to the PM by PI(3)P-independent processes [19,20]. In addition, a portion of EE membranes recruits Rab11 [20], which generates Rab11 carriers that deliver EE-derived membranes with cargo to the ERC for slow recycling back to the PM or rerouting to the TGN [21]. Thus, it is generally believed that the ERC is maintained by the incoming flow of Rab11-positive EE intermediates [12]. The development of Rab11 carriers is a PI(3)P-dependent process, which requires localized activation of class II PI3K and burst of PI(3)P production and the sites of Rab11-vesicle budding [22]. However, it is unclear how these processes are related to the overall PI(3)P production at EEs by the activity of class III PI3K, which is the master PI(3)P producer at EE membranes [17].

To explore the role of EE maturation in the biogenesis of the pre-AC, we aimed to deplete a branch of EE maturation after the establishment and the progression of the E phase of infection and before major rearrangements associated with pre-AC development occur. This was possible by the rapid depletion of class III PI3K-dependent PI(3)P production using the VPS34-IN1 (IN1) inhibitor. This recently discovered compound inhibits class III PI3K isoform without affecting the other 25 lipid kinases or more than 340 protein kinases [23,24]. IN1 depletes the quick-turnover Vps34-synthesized PI(3)P pool and decreases PI(3)P levels in the endosomes within 1 min [23]. First, we tested this tool in uninfected and unperturbed cells. We have found that in Balb/3T3 cells, depletion of the Vps34-derived PI(3)P pool abolishes recycling cargo sorting into Rab11-positive endosomes and its delivery to the ERC, in addition to expected inhibition of EE-to-LE transition. These observations in uninfected cells, which shed light on the ERC biogenesis due to the complexity of the data, are elaborated in the separate manuscript [25].

The study presented here uses the finding that rapid depletion of the Vps34-derived PI(3)P pool abolishes the membrane flow from EEs to the ERC. We tested if the Vps34-derived PI(3)P-dependent processes are essential for establishing CMV infection, biogenesis of the pre-AC in the E phase of infection, and the infectious progeny virus production. We also extended the analysis under conditions of long-term inhibition of PI(3)P-dependent functions by the dominant-negative effects of 2xFYVE and p40PX binding modules. Our study demonstrates that Vps34-derived PI(3)P is not essential for establishing MCMV infection, progression through the E phase of infection, and membranous organelle reorganization associated with the development of the pre-AC but, nevertheless, limits virus production.

## 2. Materials and Methods

### 2.1. Cell Lines, Viruses, and Infection Conditions

Cell culture, production of MCMV stocks, and infection of cells with MCMV have been performed according to standard procedures [26]. Balb/3T3 cells were obtained from American Type Culture Collection (ATCC, clone A31, ATCC CCL-163), and primary murine embryonic fibroblasts (MEFs) were generated from 17 days embryos of BALB/c mice. Balb/3T3 cells were grown in DMEM, and MEFs in minimal essential medium (MEM), supplemented with 10% (Balb/3T3) or 5% (MEF) of fetal bovine serum (FBS), 2 mM L-glutamine, 100 mg/mL of streptomycin, and 100 U/mL penicillin (all reagents from Gibco/Invitrogen, Grand Island, NY, USA). The cells were grown in Petri dishes as adherent cell lines and used for infection when 90% confluent.

The recombinant virus Δm138-MCMV (ΔMC95.15), with the deletion of the fcr1 (m138) gene [27], was regularly used for infection. For monitoring the progression of the MCMV cycle, we used C3X-GFP MCMV, recombinant virus generated on the wild-type background by insertion of GFP cassette (green fluorescent protein gene under the control of HCMV major immediate-early promoter) in front of *ie2* gene [28] and wild-type MCMV (strain Smith, ATCC VR-194).

Cells were infected at a multiplicity of infection (MOI) of 10 with an enhancement of infectivity by centrifugation [26], and the efficiency of infection was monitored by the immunofluorescent detection of the intracellular immediate-early 1 (IE1) protein, as described previously [8].

### 2.2. Antibodies and Reagents

Antibodies to membranous organelle markers and MCMV-encoded proteins were monoclonal (mAb) or polyclonal (pAb). The sources of primary antibody reagents and validation references are presented in Table 1.

VPS34-IN1 was purchased from Cayman Chemical Company (Ann Arbor, MI, USA) and dissolved in DMSO as 10 mM stock. Control cells were treated with an appropriate dilution of DMSO in the tissue-culture medium (1:1000 to 1:10,000).

Alexa Fluor (AF)^594^- and AF^555^-holo-transferrin (hTf) were from Molecular Probes (Eugene, OR). AF^488^-, AF^594^-, and AF^555^-conjugated secondary antibodies to mouse IgG_2a_, mouse IgG_1_, rat IgG, rabbit IgG, and chicken IgG were from Invitrogen Molecular Probes Thermo Ficher Scientific (Leiden, NL, USA), and AF^680^-conjugated IgG_2a_ was from Jackson ImmunoReserach Europe Ltd. (Cambridge UK). Secondary antibodies goat anti-rabbit and anti-mouse conjugated with HRP were from Jackson Laboratories (Bar Harbor, ME, USA). Propidium iodide and other chemicals were from Sigma-Aldrich Chemie GmbH (Schnelldorf, Germany).

### 2.3. Transfection of PI3P-Binding Domains 2xFYVE and p40PX

Murine Stem Cell retroviral vectors (MSCV) containing EGFP, YFP, EGFP-tagged 2xFYVE domain of Hrs (EGFP-2xFYVE), EGFP-tagged 2xFYVE domain of Hrs with a double mutation (EGFP-2xFYVE^C215S^), YFP-tagged PX domain of p40phox (YFP-PX), YFP-tagged PX domain of p40phox with a point mutation (YFP-PX^R57Q^), and EGFP-PH_PLCδ1_ were described previously [42]. Indicated constructs were used for transient transfection of Balb/3T3 fibroblast-like cells using Lipofectamine 2000 transfection reagent (Invitrogen) according to the manufacturer’s instructions.

### 2.4. Flow Cytometric Analysis of Infection

Cells were infected with C3X-GFP MCMV, and expression of GFP fluorescence signal was monitored by flow cytometry. At 0, 6, and 24 h post-infection (hpi), cells were detached by short Trypsin/EDTA treatment, washed in PBS containing 10 mM EDTA, HEPES pH = 7.2, 0.1% NaN_3,_ and 2% FCS (PBS-A), and analyzed using FACSCalibur flow cytometer (Becton Dickinson & Co., San Jose, CA, USA). Dead cells were excluded by propidium iodide (1 µg/mL), and 5000 cells were acquired. The fluorescence signal was determined as mean fluorescence intensity (MFI) after subtracting the background fluorescence (ΔMFI) established in the same cells immediately after infection.

### 2.5. Flow Cytometric Quantification of Recycling

Quantitative analysis of recycling was performed on de-adhered cells using procedures described previously [43]. Cells were washed with PBS containing 5 mM EDTA and detached by 1–2 min treatment with Trypsin-EDTA solution at 37 °C. Recycling of TfR was quantified by detecting fluorochrome-conjugated Tf loss from cells after pulse internalization [43]. Cells were incubated with Alexa Fluor 555-conjugated transferrin (Tf-AF^555^, 50 μg/mL) at 37 °C for 45 min to load intracellular compartments and washed three times to remove uninternalized cell surface-bound Tf-AF^555^. The amount of internalized Tf-AF^555^ was quantified by flow cytometry (ΔMFI_int, t=45_). The loss of fluorescence by recycling was determined after incubation at 37 °C for different periods (ΔMFI_rec, t=x_) in the medium containing 200 μg/mL of unlabeled Tf. The percentage of the recycled was calculated as (1 − ΔMFI_rec, t=x_/ΔMFI_int, t=45_) × 100.

### 2.6. Immunofluorescence and Microscopy

Cells grown on coverslips were fixed with 4% formaldehyde (20 min at r.t.) and permeabilized at 37 °C for 20 min with 0.5–1% Tween 20. After permeabilization, cells were incubated with primary Abs for 60 min, unbound Abs washed with PBS, and incubated for 60 min with an appropriate fluorochrome-conjugated secondary reagent (Table S1). After three washes in PBS, cells were embedded in Mowiol (Fluka Chemicals, Selzee, Germany)-DABCO (Sigma Chemical Co, Steinheim, Germany) in PBS containing 50% glycerol, and analyzed by epifluorescence and confocal microscopy.

The samples were analyzed using an Olympus BX52 microscope (camera DP72CCD, software CellF, magnification 400×).

Imaging was performed on an Olympus Fluoview FV300 confocal microscope (Olympus Optical Co., Tokyo, Japan) equipped with Ar 488, He/Ne 543, and He/Ne 633 lasers, or on a Leica DMI8 inverted confocal microscope (confocal part: TCS SP8; Leica Microsystems GmbH, Wetzlar, Germany) equipped with a UV laser (diode 405), Ar 488, DPSS 561, and He/Ne 633 lasers. The images were acquired using Fluoview software, version 4.3 FV 300 (Olympus Optical Co., Tokyo, Japan), PLAPO60xO objective, appropriate filters, and PMT detectors. Confocal aperture was set to 2. Otherwise, images were acquired using LAS (Leica Application Suite) X software (Leica Microsystems GmbH, Wetzlar, Germany), HC PLAPO CS2 (63x/1.40 oil) objective, and 4 detectors (2xPMT and 2xHyD). The z-series of 0.5 μm optical sections were acquired sequentially with the offset below 5% and medium scan speed (1.65 s/scan). Images (515 × 512 pixels) were captured at different zoom values (zoom factor: 0.75–6.0) with pixel size from 481.47 nm × 481.47 nm to 60.18 nm × 60.18 nm.

To avoid the crosstalk between emission spectra, we used barrier filters for the Olympus FV300 microscope. For double fluorescence, the interval was 510–530 nm for green fluorescence (AF^488^) and above 660 nm (AF^594^ and AF^680^). For triple fluorescence, the interval was 510–530 nm for green fluorescence (AF^488^), 585–640 nm for red fluorescence (AF^594^), and above 660 nm for far-red fluorescence (AF^680^). For the Leica DMI8 microscope, the detectors were set to intervals of 500–535 nm for green fluorescence, 600–630 nm for red fluorescence, and 660–695 nm for far-red fluorescence.

### 2.7. Image Analysis

The images were exported as a TIFF and analyzed using ImageJ 1.53c software and available plugins without any image rendition and additional processing. Focus plane images were used for image presentation and colocalization presentation by plotting profiles along the line.

Colocalization events were quantitatively evaluated on images with a 120.37 × 120.57 nm pixel size using a global statistic approach that performs intensity correlation coefficient-based (ICCB) analyses. We used the JACoP plugin (https://imagej.net/plugins/jacop, accessed on 21 August 2021) [44] to calculate Manders’ overlap coefficients (M1 and M2) within the entire z-stack for three-dimensional (3D) analysis of colocalization. The background was partially eliminated during the image acquisition process by adjusting detector settings to detect the maximal fluorescence intensity in red and green channels. The entire stack (12–18 confocal sections for infected cells) was imported into the ImageJ, the cell area outlined by the freehand tool and, by cropping and clearing, the fluorescence signal outside the area was eliminated. After the channel split, the stack of green and red channels was analyzed for pixel overlap using the JACoP plugin. The best-fit lower threshold to eliminate most of the signal background (Costes automatic thresholding method) was determined using the threshold tool and confirmed by visual inspection. Measures were made on 10–15 cells per experiment on the entire z-series.

Quantification of fluorescence intensity was performed on focal-plane images with a pixel size of 120.37 × 120.57 nm using Otsu Auto Threshold and Measure plugins, according to the published protocols [45]. Briefly, cells of interest were selected using the freeform selection tool, and area, integrated density, and mean gray value was measured for each selected cell. For background correction, we used the region next to selected cells that had no fluorescence. Total corrected cell fluorescence (TCCF) for each cell of interest was calculated using the formula: integrated density—(area of selected cell x mean fluorescence of background readings).

Quantification of the juxtanuclear area was performed within ϕ12 µm circle, centered on the area with the highest fluorescent signal, as described in previous studies for mannose-6-phosphate receptor trafficking [46]. The intensity inside the circle was quantified relative to the total intensity in the whole cells.

Volume Viewer plugin was used for the reconstruction of 3D images of the entire z-series. Briefly, the image sequence of the entire z-series is imported into ImageJ, and the image stack is projected using the Volume mode and Trilinear interpolation. Z-Aspect and the offset were adjusted visually to eliminate the background and distinguish the spatial distribution of labeled objects.

### 2.8. Western Blot

Cellular extracts for WB analysis were prepared in RIPA lysis buffer supplemented with protease inhibitors, separated by SDS-PAGE, and blotted onto a polyvinylidene difluoride (PVDF-P) WB membrane (Millipore) at 60 to 70 V for 1 h. Membranes were incubated with 1% blocking reagent (Roche Diagnostics GmbH, Mannheim, Germany) for 1 h, followed by 1-h to overnight incubation with primary Abs, three cycles of washing (TBS with 0.05% Tween 20 [TBS-T buffer]), and a 45-min incubation with peroxidase-conjugated secondary reagent diluted in TBS buffer containing 0.5% blocking reagent. After being washed three times with TBS-T buffer (pH 7.5), membranes were incubated for 1 min with SignalFire Elite ECL Reagent (Cell Signaling Technology, Danvers, MA, USA) and enveloped into plastic wrap. Signals were detected by Transilluminator Alliance 4.7 (Uvitec Ltd., Cambridge, UK).

Within each experiment on infected cells, we monitored infection level by detection of IE1 protein and β-actin as a control for cellular gene expression. The expression level of other viral and cellular proteins was analyzed, whenever possible, on the same membranes. Each Western blot data was reproduced under different experimental conditions at least once. The membranes were subjected to various exposures, and the exposure with the clearest background/signal ratio was used for data presentation. Western blot signals were analyzed by Image J 1.53 software and normalized to actin signal used as a loading control. We first calculated the normalization factor for every lane according to the formula: lane normalization factor = observed signal of actin for every lane/highest observed signal of actin for the blot. Following that, normalized experimental signals were calculated as the observed experimental signal/lane normalization factor ratio.

### 2.9. Fluorescent-Activated Cell Sorting

YFP-PX transfected (30–32 hrs p.t.) cells were infected with MCMV (MOI 10), and at 0, 6, and 16 h after infection were harvested by the short Trypsin-EDTA treatment. After two washes with PBS, the cells were resuspended in PBS containing 3% FCS and 2 μg/mL propidium iodide. The cell suspension was filtered through a nylon mesh, and living YFP-positive cells were sorted with FACS Aria cell sorter (Becton Dickinson) using standard procedures. Un-transfected cells were used to set gates for GFP-negative cells. Altogether, 35 thousand YFP-positive cells were harvested and resuspended in RIPA buffer containing PMSF (1:100) and protease inhibitors cocktail to prepare samples for Western blot analysis.

### 2.10. Analysis of MCMV Growth In Vitro

The duplicate cultures of Balb/3T3 cells and MEFs in 24-well plates (approx. 9 × 10^4^ cells/well) were infected with Δm138-MCMV at an MOI of 10. The inocula were removed after 4 h, the medium, containing either 3 µM IN1 or solvent (DMSO), was added, and the incubation was continued for up to four days. The supernatants of infected cultures were harvested daily on days 1 to 4 after infection. The cell cultures were washed with a cold medium, resuspended in 1 mL fresh medium, and subjected to three freezing and thawing cycles. The amounts of cell-associated infectious particles were determined by the three-replicate plaque assay on MEFs in 48-well plates [26].

### 2.11. Data Presentation and Statistics

The data are presented as the mean ± standard error of the mean (SEM). The significance of difference was tested using Student’s *t*-test and differences were considered significant when *p* values were <0.05 (* *p* < 0.05; ** *p* < 0.01; and *** *p* < 0.001).

## 3. Results

### 3.1. Enrichment of PI(3)P-Positive Membranous Domains in the Pre-AC of MCMV Infected Cells

**Figure 1:**
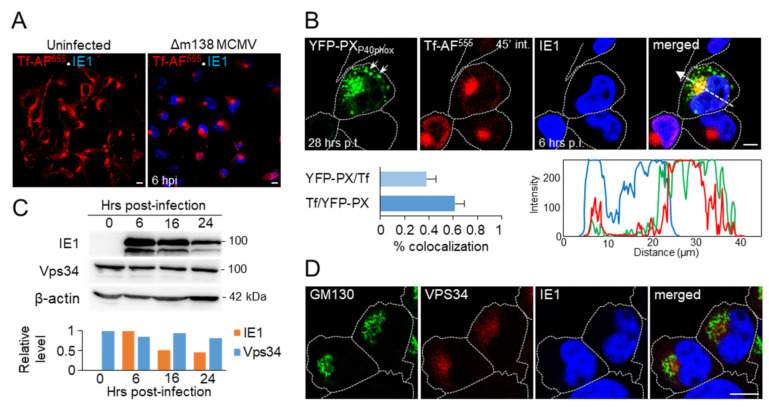
PI(3)P production and enrichment of Vps34 within the pre-AC of MCMV-infected cells. (**A**) Uninfected and Δm138-MCMV infected (MOI of 10, 6 hpi) Balb/3T3 cells were incubated for 45 min with 50 µg/mL of Tf-AF^555^, fixed, and stained with mAb against immediate-early 1 (IE1) protein of MCMV, which was visualized with AF^680^-conjugated anti-mouse IgG_1_. (**B**) Balb/3T3 cells were transfected with MSCV containing YFP-PX_P40phox_ PI(3)P-binding module and ~21 h after transfection infected with Δm138-MCMV. At 6 h after infection, the cells were incubated for 45 min with Tf-AF^555^, fixed, permeabilized, and stained against IE1 protein (blue fluorescence). Triple-stained images are shown (pixel size 240.74 × 240.74 nm; focal plane across the mid-section of the cells). Zoomed images (pixel size 120.37 × 120.37 nm) were analyzed through the entire z-stacks for colocalization using either M1/M2 coefficients of pixel overlap (left panel) or by plotting fluorescence intensity profiles along white dotted arrow lines (right panel) on MaxEntropy thresholded of images. Data represent mean ± SEM per cell (n = 10–15). (**C**) Western blot analysis of IE1, Vps34, and β-actin in the course of MCMV infection. (**D**) Triple immunofluorescence images (pixel size 180.55 × 180.55 nm) of 6 h infected cells stained with anti-GM130 (mouse IgG_1_), anti-Vps34 (rabbit IgG), and anti-IE1 (mouse-IgG_2a_), visualized with the appropriate fluorochrome-conjugated non-crossreactive secondary reagents. Cell borders are indicated by fine dotted lines. Bars, 10 μm.

Our previous studies demonstrated reorganization of the membranous system in MCMV infected cells, initiated 5–6 h after infection at MOI of 10 [8,11,47]. At 6 h post-infection (hpi), the EE system of infected cells is compacted around the cell center and accessible to the incoming endosomal flow, as demonstrated by 45 min internalization of transferrin (Figure 1A).

To visualize PI(3)P-enriched domains, we transfected cells with MSCV vector containing YFP-p40PX construct and infected with MCMV at 21 h post-transfection (p.t.). We analyzed infected cells by confocal microscopy at 27–28 h p.t. (6 hpi), when the expression level of YFP-p40PX is still relatively low, and therefore its inhibitory effect on PI(3)P-binding effectors was incomplete. As demonstrated in Figure 1B, the transfected cell displayed green fluorescence of PI(3)P-enriched vesicles, mainly localized in the 10–15 µm wide perinuclear area that imprinted the kidney-shaped nucleus. The PI(3)P-enriched vacuoles were also present at the cell periphery (Figure 1B, indicated by arrows). To show EEs within these PI(3)P-positive membranous organelles, we exposed cells to Tf-AF^555^ for 45 min, known to load EEs and the ERC, but not late endosomes (LEs). This labeling demonstrated Tf-AF^555^ accumulation at the inner areas of the YFP-p40PX-labeled cluster, indicating that EE- and the ERC-derived membranous organelles concentrate around the cell center, as observed in the neighboring un-transfected cells. Quantitative colocalization analysis demonstrated ~40% overlap of YFP-p40PX with internalized Tf-AF^555^ (Figure 1B), suggesting that ~40% of PI(3)P-positive structures are EEs. Furthermore, ~60% of internalized Tf-AF^555^ colocalized with YFP-p40PX (Figure 1B), suggesting that ~60% of internalized TfRs are retained in EEs, and the rest mainly localized in the ERC-derived membranous entities, as reported previously [11,47]. This pattern is consistent with the Tf-loading of the inner pre-AC, which accommodates numerous EE- and ERC-derived endosomal entities [8]. The PI(3)P-positive membranous organelles around the Tf-AF^555^-loaded cluster likely represent EEs or LEs, consistent with LE dislocation from the inner pre-AC area [8]. The same pattern was observed in cells expressing EGFP-2xFYVE, another PI(3)P-binding module (Figure S1A). The confinement of TF-AF^555^ to PI(3)P-positive vesicles was specific as it was not observed in cells expressing EGFP-PHPLCδ1, a construct that binds to PI(4,5)P_2_, mainly at the plasma membrane (Figure S1B). Additionally, colocalization was not observed in cells expressing 2xFYVE^C215S^ or p40PX^R57Q^ (Figure S1C,D), two constructs with mutations that abolish PI(3)P binding [48,49], and in cells expressing EGFP alone (Figure S1E). The fluorescence signal was dispersed in the cytosol and nucleus in these cells and was not membrane-associated, as described in other studies [22,50,51,52,53]

Given that more than 90% of endosomal PI(3)P is produced by the Vps34-containing class III PI3K complex [16,33,48], we analyzed Vps34 expression in the course of MCMV infection. Western blot analysis demonstrated that MCMV infection, monitored by the major immediate-early 1 (IE1) protein expression, did not change the Vps34 levels (Figure 1C). Immunofluorescence analysis at 6 hpi showed displaced GM130-positive membranous organelles representing the cis-Golgi (Figure 1D), the earliest membranous organelle reorganization event displaying the outer pre-AC [8]. Vps34-positive membranous structures mainly concentrated within the established Golgi ring representing the inner pre-AC, but also Vps34 was found on membranous organelles outside the outer pre-AC ring (Figure 1D). The observed pattern is consistent with the Vps34 distribution at EEs and LEs [51] and aligns with the above-described PI(3)P distribution in MCMV infected cells.

Altogether, these experiments demonstrate that Vps34-derived PI(3)P production, and PI(3)P-enriched membranes represent a substantial part of the pre-AC, especially at the inner pre-AC membranous organelles.

### 3.2. PI(3)P Depletion and Inhibition of PI(3)P-Associated Functions Do Not Alter the Establishment of MCMV Infection and Progression through the Early Phase of Infection

To determine the role of PI(3)P-associated functions in the pre-AC biogenesis, we have chosen two approaches: pharmacological depletion of the Vps34-derived PI(3)P pool by VPS34-IN1 (IN1) and long-term overexpression of 2xFYVE and p40PX PI(3)P-binding domains. IN1 is a potent and selective inhibitor of catalytic activity of Vps34 [23] which reorganizes the EE system, including the flow of membranes from EEs to the ERC [25]. Long-term overexpression of 2xFYVE and p40PX domains competitively saturate PI(3)P at endosomes and thereby exhibit dominant-negative effects of PI(3)P-associated functions [50,51,52,53].

**Figure 2:**
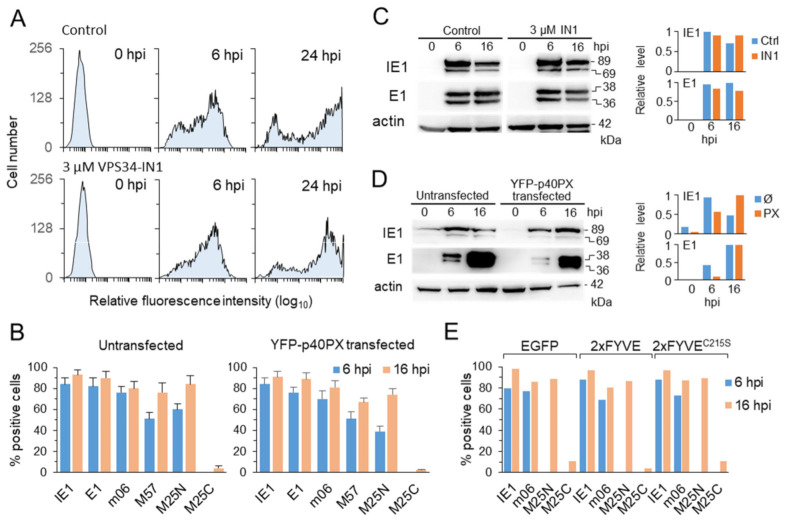
Effect of the PI(3)P depletion and PI(3)P saturation on the establishment of MCMV infection and progression through the early phase of infection. (**A**) Flow-cytometric quantification of GFP expression after infection with C3X-GFP MCMV. Infection and progression through the E phase of infection (0–16 hpi) were monitored on Balb/3T3 cells in the presence of DMSO (control) or 3 µM IN1. (**B**) Percentage of untransfected and YFP-p40PX-MSCV-transfected cells expressing immediate early (IE1), early (E1, m06, and M25N), and late (M25C) proteins of MCMV determined by immunofluorescence staining. The cells were infected with Δm138-MCMV 42 h post-transfection (MOI 10). (**C**) Western blot analysis of IE1 (68/69 kDa) and E1 (36/38 kDa), expression in cells infected with Δm138-MCMV (MOI 10) in the presence of DMSO (control) and 3 µM IN1. (**D**) Western blot analysis of IE1 and E1 expression in untransfected and YFP-p40PX-MSCV-transfected cells infected with Δm138-MCMV (MOI 10). The transfected cells were sorted with the FACS Aria cell sorter. (**E**) Percentage of EGFP-MSCV-, EGFP-2xFYVE-MSCV-, and EGFP-2xFYVE^C215S^-MSCV-transfected cells expressing MCMV proteins after infection with Δm138-MCMV (42 h post-transfection, MOI 10) determined by immunofluorescence staining.

The establishment of herpesvirus [54] and human CMV [55] infection requires intact endosomal functions; however, there are no reports on the endosomal requirement for the establishment of MCMV infection. Additionally, very little information is available about the endosomal requirement for the progression of the life cycle of CMVs. Thus, we first tested whether depletion of Vps34-derived PI(3)P pool and saturation of PI(3)P affects the establishment of MCMV infection and progression through the IE and E phases of infection. We monitored the establishment of infection in IN1-treated cells by flow cytometry using GFP-expressing recombinant virus (Figure 2A) and expression of MCMV proteins by immunofluorescence and by Western blot in IN1-treated and 2xFYVE- and p40PX-expressing cells (Figure 2B–E). The IE phase of infection was represented by the IE1 protein expressed within the first hour after infection. The E phase of infection was represented by four proteins that characterize various stages of the E phase (Figure 2B): the early 1 (E1), which is expressed 1–2 hpi [38], the m06, expressed after 2–3 hpi [41], M57, expressed after 4–6 hpi [40], and M25, expressed as 105 kDa form in the nucleus at 6–8 hpi (M25N) and 130 kDa form in the cytoplasm at 15–16 hpi (M25C) [56,57].

To monitor the establishment of infection in untreated and IN1-treated cells, we infected cells with C3X-GFP MCMV, a recombinant virus with GFP cassette under the control of HCMV major immediate-early promoter in front of *ie2* gene [28]. The expression of green fluorescence was monitored by flow cytometry 24 h after infection to cover all phases of the MCMV replication cycle. As demonstrated in Figure 2A, a similar fluorescence intensity profile was determined in untreated and IN1-treated cells after 6 hpi. This observation was confirmed on cells infected with Δm138-MCMV by immunofluorescence (data not shown, see later figures) and Western blot analysis of IE1 and E1 (Figure 2C). We did not observe a significant difference in the amounts of these proteins in IN1-treated cells, compared to untreated, at 6 h post-infection (Figure 2C). These data indicate that PI(3)P depletion does not affect virus attachment, entry, or unpackaging, the integration of virus genome and IE gene expression after initiation of infection, or the IE gene expression throughout the E phase of infection. However, at the late stages of infection (24 hpi), a shift in fluorescence profile to the left was observed, although the overall proportion of cells expressing green fluorescence remained similar (Figure 2A). These profiles suggest that PI(3)P-dependent functions may be required for the overall stability of the MCMV replication cycle at later stages of the E phase or during the late phase of infection.

To examine the role of the availability of PI(3)P domains in the E-phase of MCMV infection, we transfected cells with either EGFP-2xFYVE or YFP-p40PX and infected them at 42–48 h after transfection. At these times, most transfected cells displayed fluorescent enlarged endosomes and can be distinguished from neighboring un-transfected cells that served as an internal control. The over-expression of YFP-p40PX did not affect the establishment of the infection as determined by Western blot analysis of IE1 and E1 protein expression in flow-cytometry sorted YFP-p40PX-expressing cells (Figure 2D), or immunofluorescence quantification of IE1 and E1 expressing YFP-p40PX-positive cells (Figure 2B). Although the Western blot analysis suggests the delay of the E phase of infection (Figure 2D), YFP-p40PX expression did not affect progression through the E phase of infection, as represented by monitoring the expression of the four MCMV proteins representing various stages of the E phase (Figure 2B). The same was observed after transfection of cells with EGFP-2xFYVE, its PI(3)P-nonbinding mutated form EGFP-2xFYVE^C215S^, and EGFP alone (Figure 2E), indicating that neither inhibition of PI(3)P-associated functions nor overexpression of PI(3)P-binding domains affect the progression of the E phase of the MCMV replication cycle.

### 3.3. Depletion of Vps34-Derived PI(3)P Pool Disperses PI(3)P-Binding Proteins and Induces Similar Alterations of Endosomal System in MCMV-Infected Cells as in Uninfected Cells

**Figure 3:**
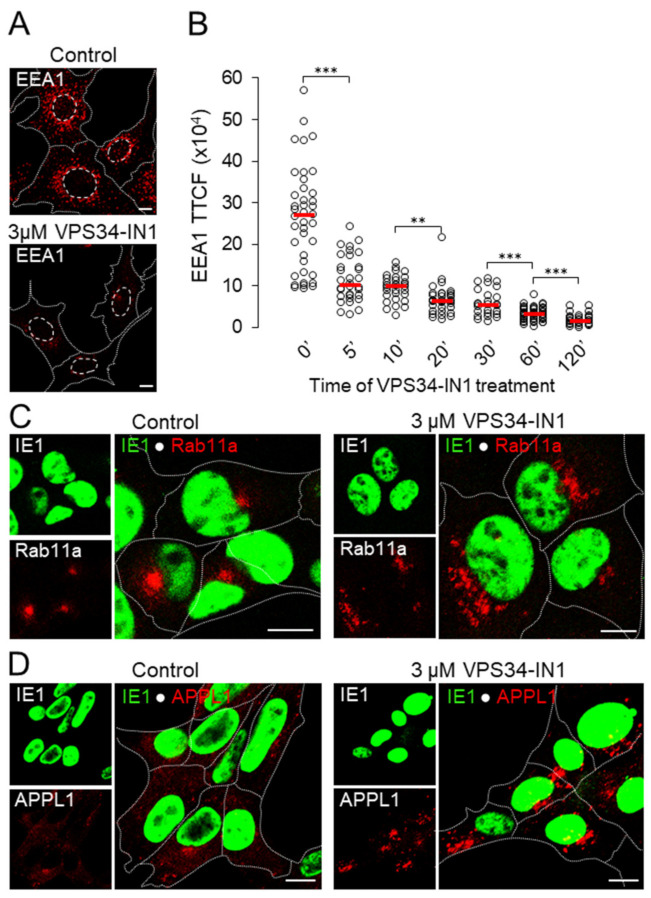
Depletion of Vps34-derived PI(3)P disperses PI(3)P-binding proteins and induces a similar set of EE alterations in MCMV infected cells as in uninfected cells. (**A**) Immunofluorescence images of EEA1 expression in control (DMSO) and 3 µM IN1-treated (60 min) Balb/3T3 cells. (**B**) Time-course of EEA1 dissociation in uninfected cells treated with 3 µM IN1. Fixed and permeabilized cells were stained against EEA1, and the Total Corrected Cell Fluorescence (TCCF) of each cell was quantified at Otsu-thresholded focal plane confocal images using ImageJ. The data represent individual cells and horizontal bars the median value. (**C**,**D**) IN1 treatment disperses Rab11-positive compartments and induces perinuclear recruitment of APPL1. Cells infected with Δm138-MCMV (MOI of 10) were treated at 4 hpi either with the solvent (DMSO) or with 3 µM IN1 for 120 min, fixed, permeabilized, and stained with rabbit Abs against Rab11a or APPL1 (red fluorescence) and mouse mAb against immediate-early 1 (IE1) protein of MCMV (green fluorescence). The appropriate non-crossreactive secondary antibodies conjugated with the AF^594^ and AF^488^ were used to visualize the reaction. Cell borders are indicated by fine dotted lines. Significance: ** = *p* ≤ 0.01, *** = *p* ≤ 0.001. Bars, 10 μm.

To deplete the Vps34-derived PI(3)P pool, we used a 3 µM concentration of the inhibitor, which preserves the cell viability for a long time, enabling long-term treatment. At this concentration, IN1 induces dissociation of FYVE- and PX-domain-containing PI(3)P-binding modules and endogenously expressed proteins (i.e., EEA1 and SNX3) from endosomes and inhibits PI(3)P-dependent phosphorylation of SGK3 [25]. To determine the effect of the inhibitor more precisely, we treated uninfected cells and monitored the time-course of EEA1 dissociation from membranes by quantifying the fluorescence intensity at EEA1 on confocal images. As shown in Figure 3A, EEA1 resembled a typical appearance of EEs as punctate perinuclear structures in control-treated (DMSO) cells, whereas, after 60 min of treatment, very little EEA1 was associated with membranes of EEs in IN1-treated cells. A significant fraction of EEA1 dissociated from EEs already after 5 min of treatment, continued with prolonged incubation and resulted in almost undetectable levels after 120 min of treatment (Figure 3B). As expected, these data demonstrate that IN1 rapidly depletes Vps34-derived PI(3)P pool from the endosome, resulting in gradual dissociation of an endogenously bound protein from endosomes. A similar result was obtained by monitoring fluorescent FYVE- and PX-domain containing PI(3)P-binding modules (data not shown) [25].

To test the effects of PI(3)P depletion on the membranous organelle reorganization in the E phase of MCMV infection, we treated cells with IN1 at 4 hpi, before cell rounding, reorganization of the Golgi, and juxtanuclear accumulation of EE-ERC-TGN interface-derived membranous organelles. After 2 h of treatment at 6 hpi, we examined whether the depletion of the Vps34-derived PI(3P) pool has a similar effect as in uninfected cells. In addition to dissociation of EEA1 (data not shown), we analyzed the expression pattern of Rab11a- and APPL1-positive membranous organelles as specific PI(3)P depletion indicators.

As we have shown previously, in uninfected cells, the 2-h IN1 treatment dispersed the ERC and initiated the development of Rab11a-positive vacuoles in the perinuclear area of the cell [25]. In MCMV-infected cells, Rab11a-positive membranes accumulated in the pericentriolar area at 6 hpi (Figure 3C), consistent with the development of the inner pre-AC [8]. However, in IN1 treated MCMV-infected cells (4–6 hpi), vacuolized Rab11a-positive membranous organelles were scattered mainly outside the pericentriolar area, indicating that IN1 induced a similar effect on EE-to-ERC trafficking as in uninfected cells. To further support this conclusion, we determined the distribution of membrane-recruited APLL1 after IN1 treatment. Namely, in untreated cells, APPL1 is mainly recruited at the cell periphery at a subset of pre-EEs, whereas after acute PI(3)P depletion, it is overrecruited to the perinuclear endosomes [25,58]. Thus, this assay is a sensitive indicator of the rapid PI(3)P depletion. In MCMV infected cells, as demonstrated in Figure 3D, APPL1 was mainly recruited at peripheral endosomes at 6 hpi and approx. 12% of the cells displayed juxtanuclear accumulation of APPL1-positive membranes. Inhibition of PI(3)P production caused the accumulation of APPL1 at perinuclear endosomes at 60–70% of infected cells (Figure 3D). As for Rab11a (Figure 3C), APPL1 was highly recruited at endosomes outside the juxtanuclear area. However, at later times of infection, the proportion of cells with APPL1 accumulation in the juxtanuclear area increased. At 16 hpi, most cells displayed a juxtanuclear aggregate of APPL1-positive membranes (data not shown), suggesting that MCMV infection itself at later time points dysregulates APPL1 membrane recruitment.

**Figure 4:**
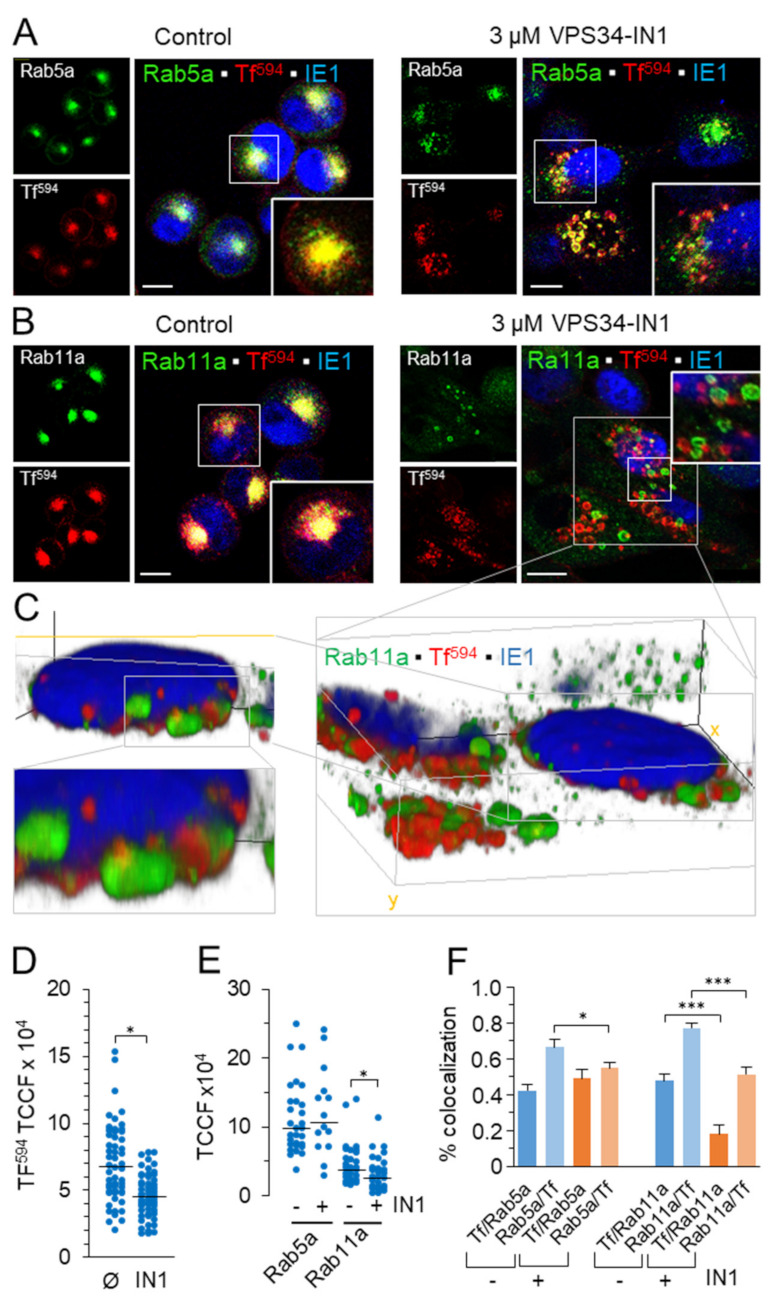
IN1 treatment prevents recycling cargo sorting into the Rab11a-dependent pathway of the recycling circuit in MCMV infected cells. Cells infected with Δm138-MCMV were treated with 3 µm IN1 or appropriate dilution of the solvent (control) at 4 hpi and exposed 45 min to Tf-AF^594^ (µg/mL) followed by staining against either Rab5a or Rab11a and IE1 proteins. Representative images of intracellular distribution of internalized Tf-AF^594^ and Rab5a (**A**) or Rab11a (**B**) relative to the nuclear distribution of the IE1 protein. Inserts present zoomed area box. Bars, 10 μm. (**C**) 3D reconstruction of the Rab11a- and Tf-AF^594^-labeled TfRs in IN1-treated MCMV infected cells. The right panel presents the 3D view of the entire z-stack of 0.3 µm sections (32 slices) from the boxed area in Figure 4B using the Image J Volume Viewer plugin. The image shown on the right presents the 3D view of the entire stack, and the images on the left give the view across the vertical section through the stack. (**D**) Quantification of intracellular Tf-AF^594^ in control (Ø) and IN1-treated cells by ImageJ analysis. Dots represent the Total Corrected Cell Fluorescence (TCCF) of individual cells in the representative experiment (N = 3) and horizontal bars the median value. (**E**) Quantification of Rab5a and Rab11a intensity in control and IN1-treated cells by ImageJ analysis. Dots represent the Total Corrected Cell Fluorescence (TCCF) of individual cells in a representative experiment (N = 3) and horizontal bars the median value (**F**). The 3D colocalization of Tf with organelle markers and organelle markers with Tf in control and IN1-treated cells based on M1/M2 coefficients of pixel overlap measured across the Costes-algorithm thresholded z-stacks of confocal images. Data represent the mean ± SEM per cell (n = 8–12). Significance: * = *p* ≤ 0.01, *** = *p* ≤ 0.001.

To test whether IN1 treatment induces similar alterations of endosomal flow in MCMV infected cells as observed in uninfected cells [25], we analyzed internalization of Tf into Rab5a- and Rab11a-positive compartments of infected cells. We treated cells with IN1 at 4 hpi and exposed cells to Tf-AF^594^ for 45 min at 6 hpi. Colocalization analysis demonstrated Tf loading into Rab5a- (Figure 4A) and Rab11a-positive (Figure 4B) endosomes in control cells. In IN1-treated cells, Tf loaded into Rab5 endosomes, which were enlarged, in some cells vacuolized and often displaced from the juxtanuclear area (Figure 4A). A similar pattern was observed in uninfected cells. The overall Tf loading was reduced (Figure 4D), suggesting that IN1 treatment also affects the receptor loading to the PM (recycling) in MCMV infected cells. The overall Rab5a recruitment was similar to that in control cells, and colocalization of Rab5a with internalized TfRs was slightly reduced (Figure 4F), presumably due to the reduced amount of Tf internalized.

As in uninfected cells, IN1-treatment enlarged, vacuolized, and displaced Rab11a-positive compartments (Figure 4B). It was challenging to define these compartments in the juxtanuclear area. The 3D imaging analysis demonstrated the segregation of TfR-loaded and enlarged Rab11a domains within the same compartment (Figure 4C). The overall signal of Rab11a was reduced in IN1-treated MCMV-infected cells (Figure 4E), as observed in uninfected cells. The Rab11a-positive compartments were not loaded with internalized TfRs (Figure 4B), as demonstrated by the very low colocalization of internalized TfRs and Rab11a (Figure 4F). These data indicate that PI(3)P depletion prevents recycling cargo loading into the Rab11a-dependent pathway and the biogenesis of the ERC also in MCMV infected cells.

Taken together, the short-term depletion of the Vps34-derived PI(3)P pool initiates a similar set of alterations at EEs as in uninfected cells, including disruption of EE-to-ERC trafficking and relocation of Rab11 endosomes.

### 3.4. Depletion of the Vps34-Derived PI(3)P Pool Does Not Prevent the Membranous Organelle Reorganization in the Early Phase of Infection

**Figure 5:**
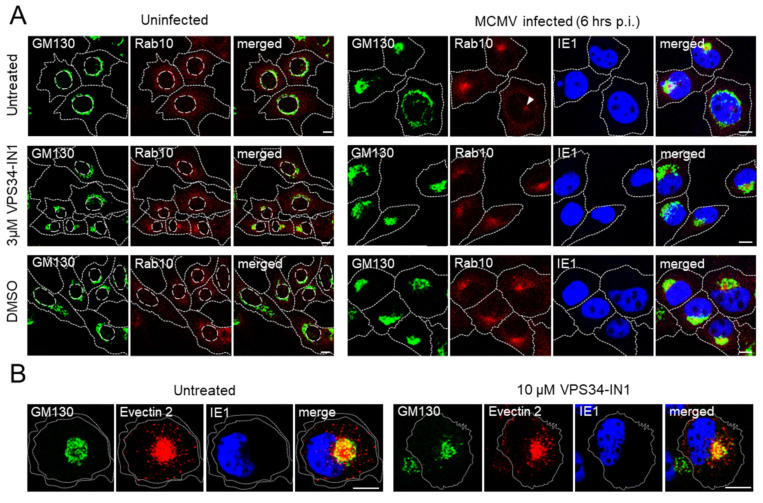
Effect of PI(3)P depletion on membranous organelle reorganization in the early phase of MCMV infection. (**A**) Representative (N = 12–15) focal-plane images of untreated, IN1-treated (3 µM), and solvent (DMSO)-treated uninfected and MCMV-infected (Δm138-MCMV, MOI 10) Balb/3T3 cells at 6 hpi. The cells were treated for 2 h starting at 4 hpi, fixed, permeabilized, and stained with mouse mAb to GM130 (IgG_1_), rabbit mAb to Rab10, and mouse mAb to IE1 (IgG_2a_), followed by non-cross reactive AF^488^-anti-mouse IgG_1_, AF^680^-anti-mouse IgG_2a_, and AF^555^-anti-rabbit IgG. (**B**) Representative images of cells subjected to the same procedure as in (**A**), treated with 10 µM IN1 instead of 3µM, and stained with rabbit polyclonal Ab against Evectin-2 (PLEKHB2) instead of anti-Rab10. Cell borders are indicated by fine dotted lines. Bars, 10 μm.

The first reorganization events of membranous organelle identified by immunofluorescence studies include dislocation of the Golgi, which establishes the outer ring of the pre-AC, and concomitant expansion of membranes derived at the EE-ERC-TGN interface [8,11]. This reorganization is clearly distinguished in approx. 40% of infected cells at 6 hpi and is present in most cells an hour later. Thus, to explore the contribution of the Vps34-derived PI(3)P in these events, we treated infected cells with IN1 at 4 hpi, the time when still no displacement of the Golgi can be observed. At 6 hpi, we stained cells with anti-GM130 antibody, which displays cis-Golgi membranes within the outer ring of the pre-AC, and anti-Rab10, which shows expanded membranes of the inner pre-AC at the EE-ERC interface [8]. Although several markers may display expansion of EE-ERC membranes within inner pre-AC, we have found that Rab10 is particularly suitable for the earliest pre-AC marker. Rab10 showed faint staining in the perinuclear area of the uninfected cells and appeared as concentrated fluorescence within the GM130-stained outer ring of the pre-AC of infected cells [8] (Figure 5A). The infection was monitored by staining the IE1 protein, which stains the entire nucleus, including nuclear indentation, as an additional sign of the reorganization of the perinuclear area. This pattern clearly distinguishes the earliest stages of pre-AC development by immunofluorescence. As exemplified in Figure 5A, the cells that compacted and reorganized the GM130-positive Golgi into the ring shape simultaneously display an accumulation of Rab10 within the ring. The infected cells that initiated the Golgi fragmentation and compacting showed initial expansion of Rab10 in the subnuclear part of the ERC (Figure 5A, indicated by arrowhead). A similar pattern was observed for Evectin-2 (data not shown) [8], which displays phosphatidyl-serine-rich outgoing membranes of the ERC.

The presence of IN1 from 4 h of infection neither prevented the Golgi compacting and displacement, nor the accumulation of Rab10 (Figure 5A) and Evectin-2 (data not shown). The established pattern was also maintained after prolonged incubation with IN1 from 4–16 h p.i. (data not shown), and after increasing concentrations of the inhibitor, as demonstrated by Evectin-2 staining in 10 µM IN1-treated cells (Figure 5B). Given that the Golgi integrity is sensitive to DMSO, the solvent used for IN1, the cells were incubated in the medium containing the same amount of DMSO in all experiments. As demonstrated in Figure 5A, the presence of DMSO did not affect alterations induced by the infection. Together, these data indicate that Vps34-derived PI(3)P-dependent flow of membranes from EEs to the ERC is not required for the biogenesis of the pre-AC.

### 3.5. Long-Term Inhibition of PI(3)P-Associated Functions Does Not Alter Initiation of Early Phase Associated Membranous Organelle Reorganization and Development of the Pre-AC

We next analyzed whether observations obtained after acute depletion of the Vps34-derived PI(3)P pool could be confirmed in cells with long-term inhibition of PI(3)P-associated functions. In our previous study [25], we demonstrated that the overexpression of 2xFYVE and p40PX binding modules exert dominant-negative effects on the PI(3)P-dependent functions resulting in alteration of the EE system in a similar way as pharmacological depletion of Vps34-derived PI(3)P. An advantage of using these PI(3)P-binding modules may be in the competitive displacement of FYVE- and PX-domain-containing proteins from the entire endosomal PI(3)P pool.

**Figure 6:**
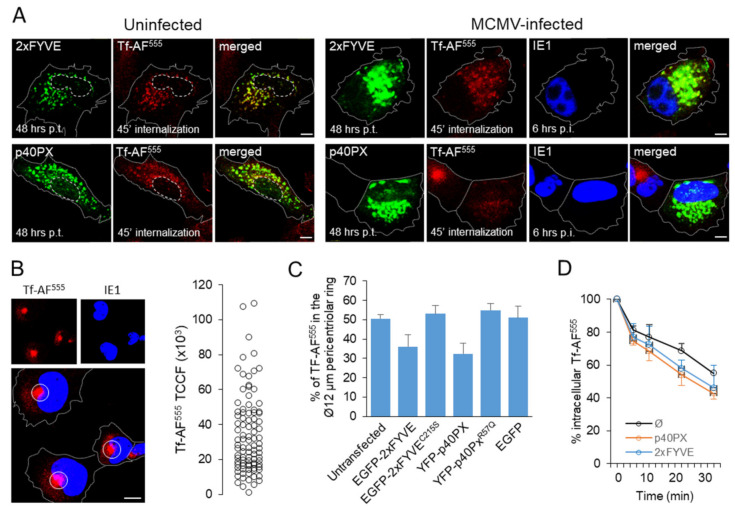
The function of EE system in 2xFYVE- and p40PX-transfected and MCMV-infected cells. (**A**) Internalization pattern of TF-AF^555^ in transfected cells. Balb/3T3 cells were transfected with EGFP-2xFYVE and YFP-p40PX, and either uninfected or MCMV infected (Δm138-MCMV, MOI 10) at 42 h p.t. After 6 h of infection (48 h p.t.) the cells were exposed to 50 µg/mL of Tf-AF^555^ for 45 min, fixed, permeabilized and stained with mAb against IE1. Cell borders are indicated by fine dotted lines and nuclei by fine dashed lines. Bars, 10 μm. (**B**) Set up for quantification of internalized TF-AF^555^ in the 12 µm juxtanuclear area representing the inner pre-AC. Images (shown on the right) of internalized Tf-AF^555^ within the 12 µm circle centered at the highest fluorescence signal were quantified using the Automatic Thresholding and Measure plugin of the ImageJ. Total Corrected Cell Fluorescence (TCCF) was calculated after subtracting the background measured in the neighboring area. The scatter plot on the right shows the distribution of TCCF in infected cells. (**C**) Mean TCCF of internalized Tf-AF^555^ in MCMV-infected and un-transfected or EGFP-2xFYVE-, EGFP-2xFYVE^C215S^-, YFP-p40PX-, YFP-p40PX^R57Q^-, and EGFP-transfected cells. (**D**) Kinetics of Tf-AF^555^ recycling in MCMV-infected and un-transfected and either YFP-p40PX- or 2xFYVE- transfected cells. The cell-associated Tf-AF^555^ was quantified by flow cytometry after gating transfected cells. Data represent the mean ± SEM of six independent experiments.

To monitor the inhibitory effect of PI(3)P-binding domains on membranous organelle reorganization, we infected cells expressing 2xFYVE and p40PX domains (~42 h p.t.) and analyzed 6 h after infection. To assess EE function, we loaded Tf-AF^555^ for 45 min and analyzed Tf-AF^555^ distribution by immunofluorescence. As shown in Figure 6A, in infected cells, internalized Tf was retained in enlarged perinuclear endosomes positive for both EGFP-2xFYVE and YFP-p40PX, as in uninfected cells. In un-transfected cells, the majority of internalized Tf-AF^555^ was concentrated in the 12 µM wide perinuclear aggregate (Figure 6B) representing the inner pre-AC [8]. Given that Tf-AF^555^ was continuously taken up over 45 min and distributed through the entire endosomal system, we quantified the relative amount of Tf-AF^555^ that loaded the 12 µM pericentriolar ring (Figure 6C). Quantification demonstrated that 50 ± 2% of internalized Tf-AF^555^ was in the inner pre-AC compartments, including EEs and ERC. In both EGFP-2xFYVE and YFP-p40PX expressing cells, the relative amount of internalized Tf-AF^555^ was lower but not absent within the 12 µM pericentriolar ring, mainly localized within enlarged EGFP-2xFYVE- and YFP-p40PX-positive endosomes. The amount of internalized Tf-AF^555^ in cells transfected with PI(3)P-binding 2xFYVE and p40PX modules or EGFP alone was similar to un-transfected cells (Figure 6C). These data demonstrate that internalized TfRs load into EEs, which accumulate in the inner pre-AC and remain within EEs, indicating the inhibition of EE-to-ERC membrane flow in MCMV-infected cells, as observed in uninfected cells. To test whether these endosomes are capable of TfR recycling, we loaded cells with Tf-AF^555^ and analyzed the loss of fluorescence by flow cytometry on gated cells expressing green fluorescence signals EGFP-2xFYVE and YFP-p40PX (Figure 6D). As observed in uninfected cells [25], the Tf-AF^555^ was released even faster from EGFP-2xFYVE- and YFP-p40PX-expressing cells than from un-transfected cells, indicating that swollen endosomes sustain the capacity of cargo recycling. The same was observed in the immunofluorescence assay (data not shown). Together, these data suggest that saturation of PI(3)P domains has similar consequences in MCMV infected cells as observed in uninfected cells.

**Figure 7:**
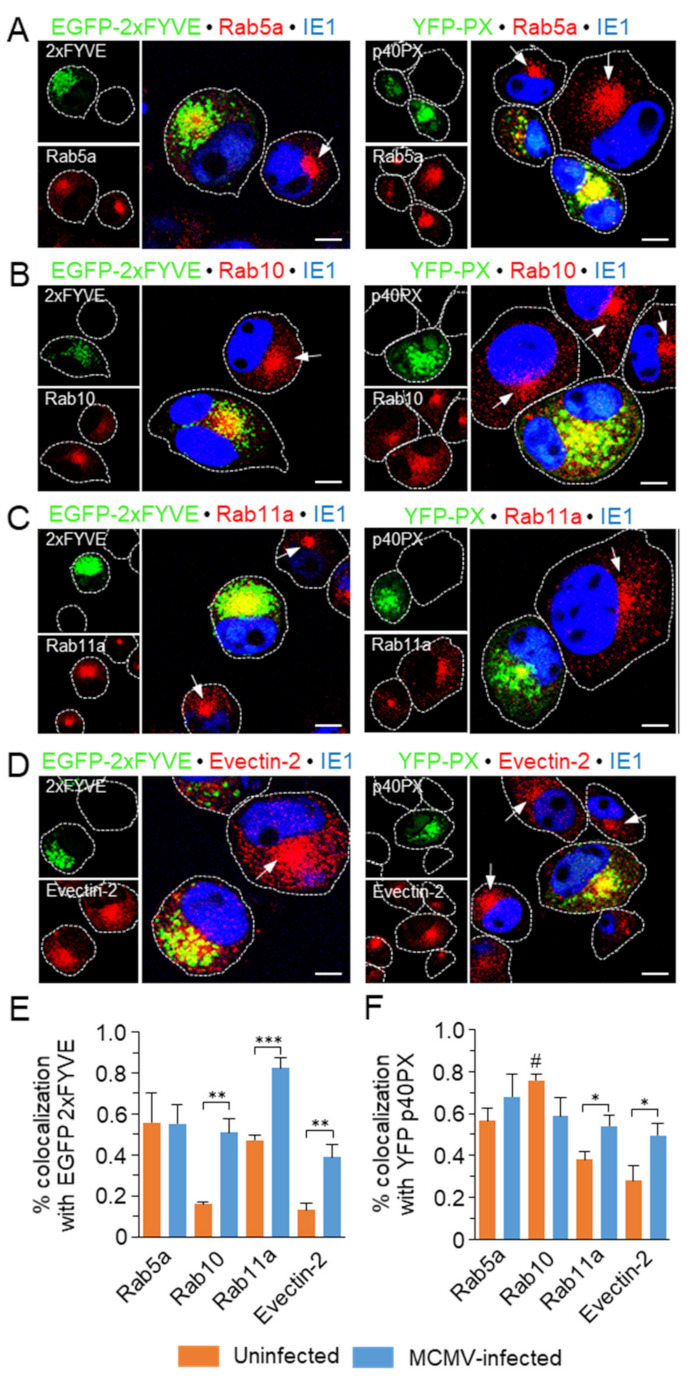
Effect of 2xFYVE and p40PX expression on the development of the inner pre-AC in MCMV infected cells. Balb/3T3 cells were transfected with EGFP-2xFYVE and YFP-p40PX and MCMV infected (Δm138-MCMV, MOI 10) at 42 h p.t. After 6 h of infection (48 h p.t.), the cells were fixed, permeabilized, and stained with mAb against IE1 and either rabbit polyclonal (**A**) anti-Rab5a, (**B**) anti-Rab10, (**C**) anti-Rab11a, or (**D**) anti-Evectin-2, followed by non-cross reactive AF^680^-anti-mouse IgG_2a_ and AF^594^-anti-rabbit IgG. Arrows point to the juxtanuclear accumulation in un-transfected cells. Cell borders are indicated by fine dotted lines. Bars, 10 μm. The 3D colocalization of Rab5a, Rab10, Rab11a, and Evectin-2 with (**E**) EGFP-2xFYVE or (**F**) YFP-p40PX in uninfected (orange bars) and MCMV-infected (blue bars) cells, based on M1/M2 coefficients of pixel overlap measured across the Costes-algorithm thresholded z-stacks of confocal images. Data represent the mean ± SEM per cell (n = 8–12). Significance: * = *p* ≤ 0.05, ** = *p* ≤ 0.01, *** = *p* ≤ 0.001.

To further monitor the effect of long-term PI(3)P saturation on membranous organelle reorganization in the E phase of MCMV infection, we performed immunofluorescence analysis of markers of EE-ERC interface in EGFP-2xFYVE- and YFP-p40PX-expressing cells. In these cells, Rab5a-positive membranes accumulated in the juxtanuclear area (Figure 7A) as in untransfected cells (Figure 7A, arrows). More than half of Rab5a colocalized with 2xFYVE (Figure 7E) and p40PX (Figure 7F), except the central part of the juxtanuclear aggregate. Similar results were observed with Rab10, which is poorly recruited to membranes of uninfected cells (Figure 5A), but highly recruited to the inner pre-AC of non-transfected (Figure 7B, arrows) as well as 2xFYVE and p40PX transfected MCMV-infected cells (Figure 7B). Little Rab10 colocalized with 2xFYVE in uninfected cells, whereas the degree of colocalization increased to more than half of overrecruited Rab10 in infected cells (Figure 7E). Interestingly, the overexpression of p40PX domains in uninfected cells resulted in an almost complete overlap of Rab10 with p40PX (Figure 7F, labeled with #), suggesting distinct relation of 2xFYVE- and p40PX-saturated EE functions with the recruitment of Rab10. However, there is no sufficient information in the literature to explain this finding. As expected, Rab10 highly overlapped with p40PX-decorated endosomes of the preAC (Figure 7F), to a similar extent as 2xFYVE. To gain more relevant colocalization information, we also analyzed the overlap of signals by Pearson’s correlation. The Pearson’s correlation of 2xFYVE and p40PX overlap with Rab10 in uninfected cells (0.147 ± 0.015 and 0.763 ± 0.045, respectively) and infected cells (0.53 ± 0.026 and 0.561 ± 0.062, respectively) suggest that the overlaps are attributed to specific rather than random colocalization. Nevertheless, the colocalization analysis demonstrated that a significant fraction of Rab10 was recruited outside the PI(3)P-containing membranous domains.

Rab11a was highly recruited in 2xFYVE- and p40PX-expressing cells (Figure 7C) and highly colocalized, especially with 2xFYVE (Figure 7E,F), suggesting that overexpression of PI(3)P-binding modules reduce segregation of Rab11a from PI(3)P-positive membranes. Evectin-2, which is also poorly recruited at membranes of uninfected cells [8] did not highly colocalize with 2xFYVE and p40PX in transfected cells (Figure 7E,F). However, the extent of colocalization significantly increased in MCMV-infected cells (Figure 7E,F), suggesting that Evectin-2 recruitment may also occur at PI(3)P membrane domains and that the inhibition of PI(3)P-associated functions interfere but does not prevent the extensive tubulation of the ERC membranes. These data demonstrate that PI(3)P binding module overexpression does not prevent the EE reorganization sequence in the E phase of infection, and suggests that the inner pre-AC contains a substantial fraction of PI(3)P-independent EE domains.

Altogether, despite some alterations of membrane flow from EEs after overexpression of PI(3)P binding modules, the analysis in MCMV infected cells suggests that PI(3)P membrane domains are not essential for the development of the pre-AC compartment.

### 3.6. Long-Term Depletion of Vps34-Derived PI(3)P Inhibits Virus Growth

**Figure 8:**
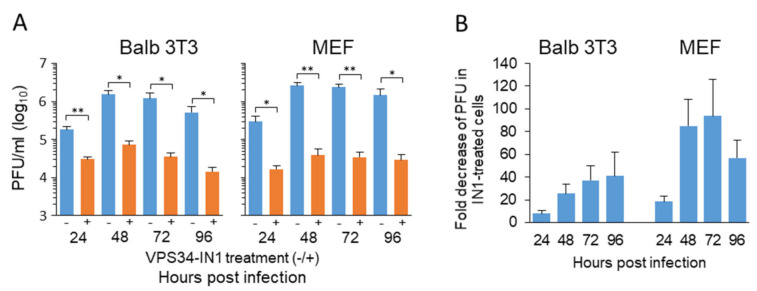
Long-term depletion of Vps34-derived PI(3)P inhibits virus growth. (**A**) Balb/3T3 cells and murine embryonal fibroblasts (MEF) were infected at an MOI of 10 with Δm138-MCMV in the absence (−) or presence (+) of 3 µM IN1. The development of infectious viral progeny was quantified under multistep growth conditions. The cell-associated viruses were determined at day 1–4 by three-cycle freezing/thawing of cells resuspended in a 1 mL medium and the standard plaque assay. (**B**) Fold decrease in PFU in IN-1 treated cell. The data represent the mean ± SEM of six experiments on Balb/3T3 cells and three experiments on MEF. Significance: * = *p* ≤ 0.05, ** = *p* ≤ 0.01.

Given that the analysis of the E phase events demonstrated that the depletion of Vps34-derived PI(3)P does not prevent the development of the pre-AC, we tested whether the absence of Vps34-derived PI(3)P affects virus growth. We have shown that similar alterations induced by the short-term could also be observed after long-term (after 24 hrs) treatment with IN1 [25]. Therefore, we monitored the quantity of cell-associated infectious virions in the presence (3 uM IN1 at 4 hpi) and the absence of the inhibitor. The first point was at 24 hpi, when we expected the earliest infectious progeny in the infected cell population, and continued monitoring at 48, 72, and 96 hpi. As demonstrated in Figure 8, the infectious virions were detected already at 24 hpi. The number of infectious virions detected at that time demonstrated roughly 2 PFU per infected cells, corresponding to approx. 50 of MCMV genomes per cell [59]. At 48 hpi, the amount of cell-associated infectious virions increased almost tenfold (17 PFU/cell), remained at a similar level at 72 hpi, and decreased to 6 PFU/cell at 96 hpi. In IN1-treated cells, the amount of detected cell-associated infectious virions was approx. 6-fold lower (0.33 PFU/cell) at 24 hpi, 20.7-fold lower (0.815 PFU/cell) at 48 hpi, 37.17-fold lower at 72 hpi, and 35.34-fold lower at 96 hpi (Figure 8A,B). These data indicate that the depletion of Vps34-derived PI(3)P dramatically attenuates the production of infectious virions. We repeated the same experiment on MEFs, primary cells most often used to analyze the virus growth, to reproduce these data. These cells were even more sensitive to the IN1 treatment, resulting in the 17.7-fold decrease in infectious virion production in IN1-treated cells at 24 hpi, 67.8-fold reduction at 48 hpi, and 69.2-fold reduction at 72 hpi, and 50-fold reduction at 96 hpi (Figure 8B). These data confirmed the results obtained in Balb/3T3 cells and demonstrate that in the absence of Vps34-derived PI(3)P, the infected cells have significantly reduced the capacity of assembly of infectious virions.

## 4. Discussion

This study explored the contribution of endosomal PI(3)P in the pre-AC of MCMV infected cell and endosomal PI(3)P-associated functions in its biogenesis. We used IN1, a rapidly acting pharmacological inhibitor of PI(3)P production [23], and 2xFYVE- and p40PX-domains that saturate endosomal PI(3)P when overexpressed and exhibit a dominant-negative effect on PI(3)P-associated functions [50,51,52,53]. IN1 acts on Vps34, an essential component of EEs recruited by Rab5, which produces PI(3)P and thereby drives EE maturation [16,17]. The short-term treatment with IN1 enabled rapid depletion of Vps34-derived PI(3)P at a time-critical for pre-AC development without interfering with other earlier events in MCMV cycle progression. IN1 also enabled prolonged long-term treatment and monitoring of MCMV cycle progression in the absence of Vps34-derived PI(3)P. Expression of fluorescently tagged FYVE and PX PI(3)P-binding modules enabled spatial and temporal monitoring of PI(3)P organelles when expressed at lower levels and exert long-term dominant-negative effects on PI(3)P associated functions due to the competitive saturation of PI(3)P at endosomes. Expression of PI(3)P-binding modules demonstrated the abundant presence of PI(3)P on endosomes that build the inner pre-AC. Both approaches showed that the PI(3)P domain is not essential for the initial stage of the AC biogenesis. However, Vps34-derived PI(3)P-pool was essential for the later virus growth.

The pre-AC development is initiated in the E phase of infection, both in HCMV [2,3,60,61] and MCMV [7,8,11,47] infected cells. A study on HCMV [61] demonstrated that Golgi fragmentation is an integral step in AC biogenesis, and our recent reports on MCMV [8,11] identified that the Golgi unlinking associated with the EE compaction and expansion of EE-ERC-TGN intermediates in the central area of the cell, could be the first events of membranous organelle reorganization. Thus, to understand the pre-AC biogenesis, it is essential to establish whether the Golgi unlinking is an initial event that results in the dysregulation of the linker compartments (ERGIC, ERC, and TGN) [62] or dysregulation of the trafficking at the EE-ERC-TGN interface leads to expansion of intermediates around the cell center which cause Golgi unlinking and its displacement.

Several studies on HCMV [63,64,65,66,67] and MCMV [7,8,47,68] demonstrated alteration in the vacuolar EE domain within the AC, known to be regulated by PI(3)P-associated functions. These alterations are manifested as vacuolization of EEs [11,47], retention of clathrin-dependent and clathrin-independent cargo [11,47,63], over recruitment of components of the VPS pathway (i.e., Hrs, PIKFyve, Vps24) [8], and delay in the EE-to-LE trafficking route [11,47]. However, there is little evidence on the relation of these alterations with PI(3)P production at EEs. The expression of Vps34 and endosome association of fluorescently labeled 2xFYVE and p40PX domains in MCMV infected cells, presented in this study, suggests that PI(3)P production at EE membranes is indistinguishable to uninfected cells and that these alterations may be connected with the dysregulation of PI(3)P-associated functions. An observation that EEA1 was transiently depleted from EEs in the E phase of infection and over recruitment of APPL1 at the perinuclear EEs of MCMV infected cells indicate that PI(3)P-dependent recruitment of effector proteins at EEs may also be dysregulated by the infection. Further studies are required for the dissection of multiple alterations of EE functions observed in CMV infected cells.

In addition to the vacuolar domain, the pre-AC development involves alteration of the tubular domain of EEs, the ERC, and the EE-ERC interface [8]. The inner area of the AC accumulates expanded tubular membranous elements, as indicated by the over recruitment of several small GTPases that regulate this interface (i.e., Rab8, Rab10, Rab11a, Rab15, Rab22a, Arf1, Arf3, and Arf6), and effector proteins associated with the tubulation at EE and ERC subdomains (i.e., EHDP1, Evectin2, and Dynamin2) [7,8,11]. These reorganizations of the tubular domain are associated with the delay in endosomal recycling of clathrin-dependent and clathrin-independent cargo in both HCMV [6,63,68] and MCMV [47] infected cells.

The development of tubular endosomal domains involves switching from PI(3)P to another phospholipid and phosphoinositide composition. Thus, most observed reorganizations in MCMV infected cells are associated with the post-PI(3)P endosomal membranes. The switch from Rab5-/PI(3)P-positive membranes towards the tubular subdomains involves replacing Rab5 with other GTPases. Among the eight Rab subfamily members that localize at tubular endosomes, Rab10 and Rab22a are essential for tubulation of endosomal membranes that sort clathrin-independent cargo [69,70], whereas Rab11a plays a key role in the segregation of clathrin-dependent cargo (i.e., transferrin receptor) [22,71]. These small GTPases are associated with membranes with lipid composition other than PI(3)P [70,72], indicating that their recruitment and activation are associated with the PI conversion. Although the conversion mechanism of Rab5a-to-Rab11a is not fully elucidated, recent studies suggest that activation of Rab11a at endosomes requires localized production of PI(3)P by the class-II PI3K [22] and recycling cargo sorting depends on the background PI(3)P levels at EEs generated by the Vps34 activity [71]. Our accompanying study [25] demonstrates that Rab11a recruitment at peripheral Ees and segregation of Rab11 endosomes do not require Vps34-derived PI(3)P. However, Rab11 endosomes generated in the absence of Vps34-derived PI(3)P cannot sort TfRs and provide its delivery to the ERC, leading to the depletion of Rab11 membranes and TfRs from the ERC. Thus, Vps34-derived PI(3)P and PI(3)P-associated functions seem essential for clathrin-dependent cargo routing towards the ERC and maintaining the Rab11a subdomain of the ERC. In the absence of Vps34-derived PI(3)P, this subdomain is depleted, and Rab11a recruitment shunted towards another Rab11a membranous domain organized at the cell periphery, as observed in cells with micro-surgically removed ERC [73]. The depletion of Vps34-derived PI(3)P induced a similar set of changes in MCMV infected cells, indicating that the infection does not alter this shunting pathway. Nevertheless, despite the reduced loading of the Rab11a subdomain of the ERC, and relocation of Rab11 endosomes to the periphery after PI(3)P depletion, the expansion of Rab10- and Evectin-2-positive membranes at the inner pre-AC was not inhibited. These data demonstrate that the expansion of the EE-ERC interface within the inner pre-AC does not depend on the availability of PI(3)P and Rab11a-associated pathways and indicates that PI(3)P-associated functions are not essential for biogenesis of the pre-AC.

Additionally, these data indicate that Rab10 recruitment is a process that does not depend on Vps34-derived PI(3)P-associated functions. It has been shown that Rab10 could be activated at Rab5 endosomes and that Rab5 and Rab10 may establish the feedback loop at EEs [74]. Rab10 can also be activated downstream in the cascades that occur at the ERC. In Balb/3T3 cells, endogenous Rab10 is recruited to membranes in the perinuclear area; however, this recruitment in most cells generates a dim immunofluorescence signal. This is consistent with the high-speed live-cell imaging of Madin–Darby Canine Kidney (MDCK) epithelial cells, which demonstrated the highly dynamic vesicular trafficking of Rab10-GFP on rapidly moving vesicles difficult to detect in static images [75]. The visualization of endogenous Rab10 on HeLaM cells, which develop long endosomal tubular extensions, demonstrated the Rab10 role in tubulation. In Balb/3T3 cells, these Rab10-decorated tubules were rarely observed, indicating a high turnover of Rab10 at membranes. However, in MCMV infected cells, this turnover appears to be significantly altered, resulting in the accumulation of Rab10 membranes within the inner pre-AC. Thus, Rab10 is particularly suitable for visualization of the inner pre-AC due to the contrast in signal between uninfected and infected cells and, together with the Golgi unlinking and displacement, can be monitored as one of the earliest events in membranous organelle reorganization that distinguishes the development of the pre-AC.

Furthermore, the over-activation of Rab10 at the inner pre-AC membranes may be a clue in the identification of CMV-targeted host-cell function that leads to extensive dysregulation of the EE-ERC interface. The Rab10 over-activation may result from increased activity of a proximal Rab10 GEF or poor recruitment and activation of a distal Rab10 GAP or Rab10 GDP. Given that the over-recruitment of Rab 10 at the inner pre-AC is associated with over recruitment of several host-cell factors linked to the membranous organelle tubulation, it is more likely that events distal of the Rab10, including regulatory cascades that shut off Rab10 activity, are altered with MCMV infection, and represent a basis for the development of the pre-AC. The over-recruitment of Evectin-2, a host-cell protein known to be recruited at phosphatidylserine of outgoing membranes of the ERC, is consistent with this [76]. This protein is also a suitable marker of the earliest pre-AC rearrangement as it is poorly recruited to membranes of the ERC in uninfected cells [8].

Our study demonstrates that PI(3)P domains build a significant part of the inner pre-AC and focuses on PI(3)P domains in the earliest stages of the pre-AC biogenesis. However, it cannot be excluded that PI(3)P and its effector proteins are essential for further steps in the pre-AC biogenesis and its maturation into the AC. After initial reorganization at 5–6 hpi [77], the pre-AC continues maturation throughout the E phase of infection and later, after viral DNA replication [8]. Therefore, further studies are required to determine the requirement of PI(3)P domains in the development and maturation of the AC. EEs also remain in the inner AC of both MCMV [8] and HCMV [64,65,67] infected cells throughout the late phases of infection, at a stage of virus secondary envelopment and egress, suggesting that EE-derived membranes may contribute to the terminal stages of virus production. Of particular significance in the envelopment may be the utilization of PI(3)P domains at EEs for budding nascent capsids into the lumen of vacuolar endosomes. This process, known as reverse-topology budding [78], is essential for vacuolar endosome maturation and formation of multivesicular endosomes in uninfected cells, and may be exploited by CMVs during the capsid envelopment. This stage of CMV maturation requires inward budding processes that are dependent on the PI(3)P and PI(3)P-to-PI(3,5)P2 switch at EE membranes. Although these steps were addressed in several studies of HCMV envelopment [79,80,81], the role of EE PI(3)P domain in CMV envelopment remains unclear. Endosomal PI(3)P domains may also be required for CMV egress, as recent studies suggest the use of the exosomal pathway for HCMV exit [81,82], which is functionally linked to the downstream maturation of LEs.

The present study demonstrates that Vps34-derived PI(3)P-associated functions are essential for efficient virus growth in infected cells. However, these functions are not required for the reorganization of the membranous system and the development of the site for the secondary envelopment. The profound effect on the virus growth presented in this study, and the results of our study, which is underway, indicate that PI(3)P-dependent signaling processes may be essential for replication of the viral DNA. For example, Vps34-derived PI(3)P also controls the serum- and glucocorticoid-regulated kinases 3 (SGK3) signaling events that regulate cell proliferation and survival [83]. The SGK3 signaling pathway may be an essential slot in the signaling cascades exploited by cytomegaloviruses to enable viral DNA replication within the environment of blocked host-cell DNA replication. Thus, it would be essential to test whether the same effects may be achieved in HCMV infected cells, which can open the potential use of VPS34-IN1 in the HCMV treatment. Given that pharmacological PI(3)P depletion alters replication of the SARS-CoV-2 virus [84,85], it is of outstanding importance to clarify the potential contribution of PI(3)P in virus replication in general.

## 5. Conclusions

PI(3)P-associated processes in the reorganization of the membranous system and cellular signaling during herpesvirus infection are insufficiently understood. Our study demonstrates that the endosomal PI(3)P domains are not required for the initial steps in the biogenesis of the AC, but intact PI(3)P-associated pathways are essential for the formation of new virions. Given that very little is known about PI(3)P-dependent pathways in herpesvirus replication, CMV, as the expert cell biologist [86], may help in this clarification.

## Figures and Tables

**Table 1:** List of antibody reagents used in this study.

Target	Reagent (Reference)
Rab5a	Rabbit mAb (Cell Signaling, Danvers, MA, USA, Cat.No. 3547) [29]
Rab10	Rabbit mAb (Cell Signaling, Danvers, MA, USA, Cat.No. 8127) [30]
Rab11a	Rabbit mAb (Cell Signaling, Danvers, MA, USA, Cat.No. 5589) [31]
APPL1	Rabbit mAb (Cell Signaling, Danvers, MA, USA, Cat.No. 3858) [32]
EEA1	Chicken pAb (Invitrogen, Thermo Fisher Scientific, Waltham, MA, USA, Cat.No. 40-5700) [33]
Evectin-2	Rabbit pAb (Biorbyt, Cambridge, UK, Cat.No. orb312792)
Vps34	Rabbit mAb (Cell Signaling, Danvers, MA, USA, Cat.No. 4263) [34]
GM130	Mouse mAb IgG_1_ (BD Biosciences, Franklin Lakes, NJ, USA, Cat.No. 610823) [35]
Actin	Mouse monoclonal (Millipore, Billerica, MA, USA, Cat.No. MAB1501) [36]
m123/IE1	Mouse mAb IgG_1_; clone CROMA 101 (University of Rijeka Center for Proteomics, Cat.No. HR-MCMV-08) [37] Mouse mAb IgG_2a_; clone IE1.01. (University of Rijeka Center for Proteomics, Cat. No. HR-MCMV-12) [38]
M112-113/E1	Mouse mAb IgG_1,_ clone CROMA 103 (University of Rijeka Center for Proteomics, Cat.No. HR-MCMV-07) [37]
M25	Mouse mAb IgG_1,_ clone M25C.01 (University of Rijeka Center for Proteomics, Cat.No. HR-MCMV-03) [39]
antiM57	Mouse mAb, clone M57.02 (University of Rijeka Center for Proteomics, Cat.No. HR-MCMV-6) [40]
m06	Mouse mAb IgG_1_, clone CROMA229 (University of Rijeka Center for Proteomics, Cat.No. HR-MCMV-02) [41]

## Data Availability

The data presented in this study are available on request from the corresponding author.

## Supplementary Materials

The following are available online at https://www.mdpi.com/article/10.3390/life11080859/s1, Table S1. Primary and secondary antibody reagents used in the immunofluorescence studies. Figure S1; Visualization of PI(3)P and PI(4,5)P membrane domains in uninfected and MCMV-infected cells. Figure S2; Original raw blots and unedited ECL images of IE1, Vps34, and β-actin used as a representative Western blot in Figure 1C of the manuscript. Figure S3; Original raw blots and unedited ECL images of IE1, E1, M25, and β-actin used as a representative Western blot in Figure 2C of the manuscript. Figure S4; Original raw blots and unedited ECL images of IE1, E1, and β-actin used as a representative Western blot in Figure 2D of the manuscript.
