# Supplementary material for: Endosomal Phosphatidylinositol-3-Phosphate-Associated Functions Are Dispensable for Establishment of the Cytomegalovirus Pre-Assembly Compartment but Essential for the Virus Growth
Source: Life (Basel). 2021 Aug 22;11(8):859. doi: 10.3390/life11080859 (PMC8398575; doi:10.3390/life11080859)
Supplement: Supplementary file 1 [file life-11-00859-s001.zip › life-1321394-supplementary.pdf]

Supplementary material

Table S1. **Primary and secondary antibody reagents used in the immunofluorescence studies**

Target	Primary antibodies	Secondary antibodies
**Rab5a**	Rabbit mAb (Cell Signaling, Cat.No. 3547)	Goat anti-rabbit IgG (H+L) cross-adsorbed secondary antibody, Alexa Fluor 488 (Thermo Fischer Scientific, Cat. No. A-11008)<br><br>Goat anti-rabbit IgG (H+L) highly cross-adsorbed secondary antibody, Alexa Fluor 594 (Thermo Fischer Scientific, Cat. No. A-11037)
**Rab10**	Rabbit mAb (Cell Signaling, Cat.No. 8127)	Goat anti-rabbit IgG (H+L) highly cross-adsorbed secondary antibody, Alexa Fluor 594 (Thermo Fischer Scientific, Cat. No. A-11037)
**Rab11a**	Rabbit mAb (Cell Signaling, Cat.No. 5589)	Goat anti-rabbit IgG (H+L) cross-adsorbed secondary antibody, Alexa Fluor 488 (Thermo Fischer Scientific, Cat. No. A-11008)<br><br>Goat anti-rabbit IgG (H+L) highly cross-adsorbed secondary antibody, Alexa Fluor 594 (Thermo Fischer Scientific, Cat. No. A-11037)
**APPL1**	Rabbit mAb (Cell Signaling, Cat.No. 3858)	Goat anti-rabbit IgG (H+L) highly cross-adsorbed secondary antibody, Alexa Fluor 594 (Thermo Fischer Scientific, Cat. No. A-11037)
**EEA1**	Chicken pAb (Invitrogen, Cat.No. 40-5700)	Goat anti-chicken IgY (H+L) secondary antibody, Alexa Fluor 555 (Thermo Fischer Scientific, Cat. No. A-21437)
**Evectin-2**	Rabbit pAb (Biorbyt, Cat.No. orb312792)	Goat anti-rabbit IgG (H+L) highly cross-adsorbed secondary antibody, Alexa Fluor 594 (Thermo Fischer Scientific, Cat. No. A-11037)
**Vps34**	Rabbit mAb (Cell Signaling, Cat.No. 4263)	Goat anti-rabbit IgG (H+L) highly cross-adsorbed secondary antibody, Alexa Fluor 594 (Thermo Fischer Scientific, Cat. No. A-11037)
**GM130**	Mouse mAb IgG_1 (BD Biosciences, Cat.No. 610823)	Goat anti-mouse IgG1 cross-adsorbed secondary antibody, Alexa Fluor 488 (Thermo Fischer Scientific, Cat. No. A-21121)
**m123/IE1**	Mouse mAb IgG_1; clone CROMA 101 (University of Rijeka Center for Proteomics, Cat. No. HR-MCMV-08)<br><br>Mouse mAb IgG_{2a}; clone IE1.01. (University of Rijeka Center for Proteomics, Cat. No. HR-MCMV-12)	Goat anti-mouse IgG1 cross-adsorbed secondary antibody, Alexa Fluor 488 (Thermo Fischer Scientific, Cat. No. A-21121)<br><br>Goat anti-mouse IgG2a AffiniPure Fab fragment, Fcγ fragment specific, Alexa Fluor 680 (Jackson ImmunoReserach Europe Ltd., Cat. No. AB_2632551)

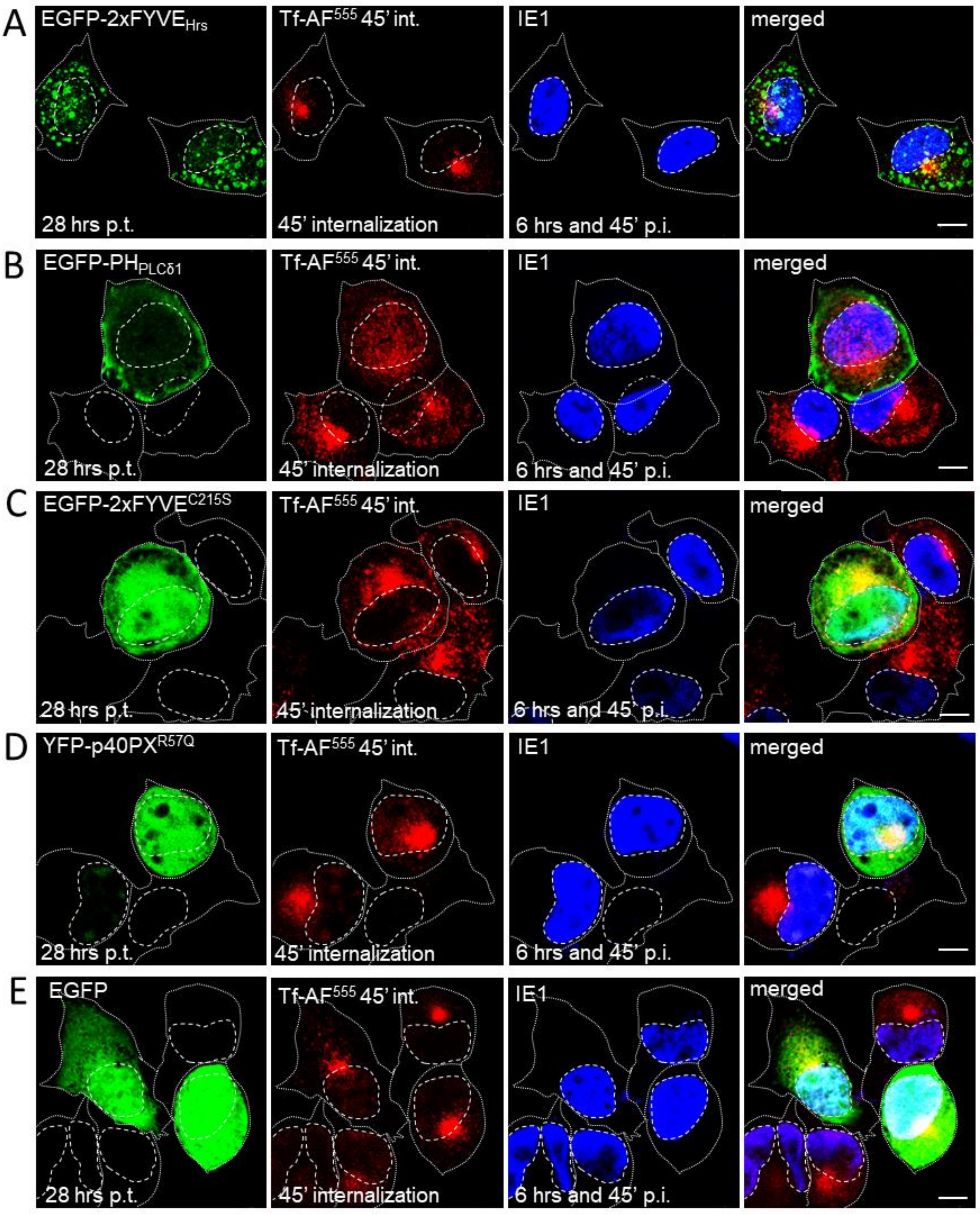

Figure S1. **Visualization of PI(3)P and PI(4,5)P2 membrane domains in uninfected and MCMV-infected cells (***related to Figure 1B***)**. Balb 3T3 cells were transfected with MSCV either containing EGFP-2xFYVE$_{Hrs}$ (**A**), or EGFP-PH$_{PLC\delta1}$ (**B**), or mutated forms EGFP-2xFYVE^{C215S} (**C**) and YFP-p40PX^{R57Q} (**D**), or EGFP only (**E**), and 21 hrs after transfection infected with Δm138-MCMV. Six hrs after infection, the cells were incubated for 45 min with Tf-AF^{555}, fixed, permeabilized, and stained against IE1 protein (blue fluorescence). Shown are the triple-stained images (pixel size 240.74x240.74 nm; focal plane across the mid-section of the cells). Cell borders are indicated by fine dotted lines and nuclei by fine dashed lines. Bars, 10 μm.

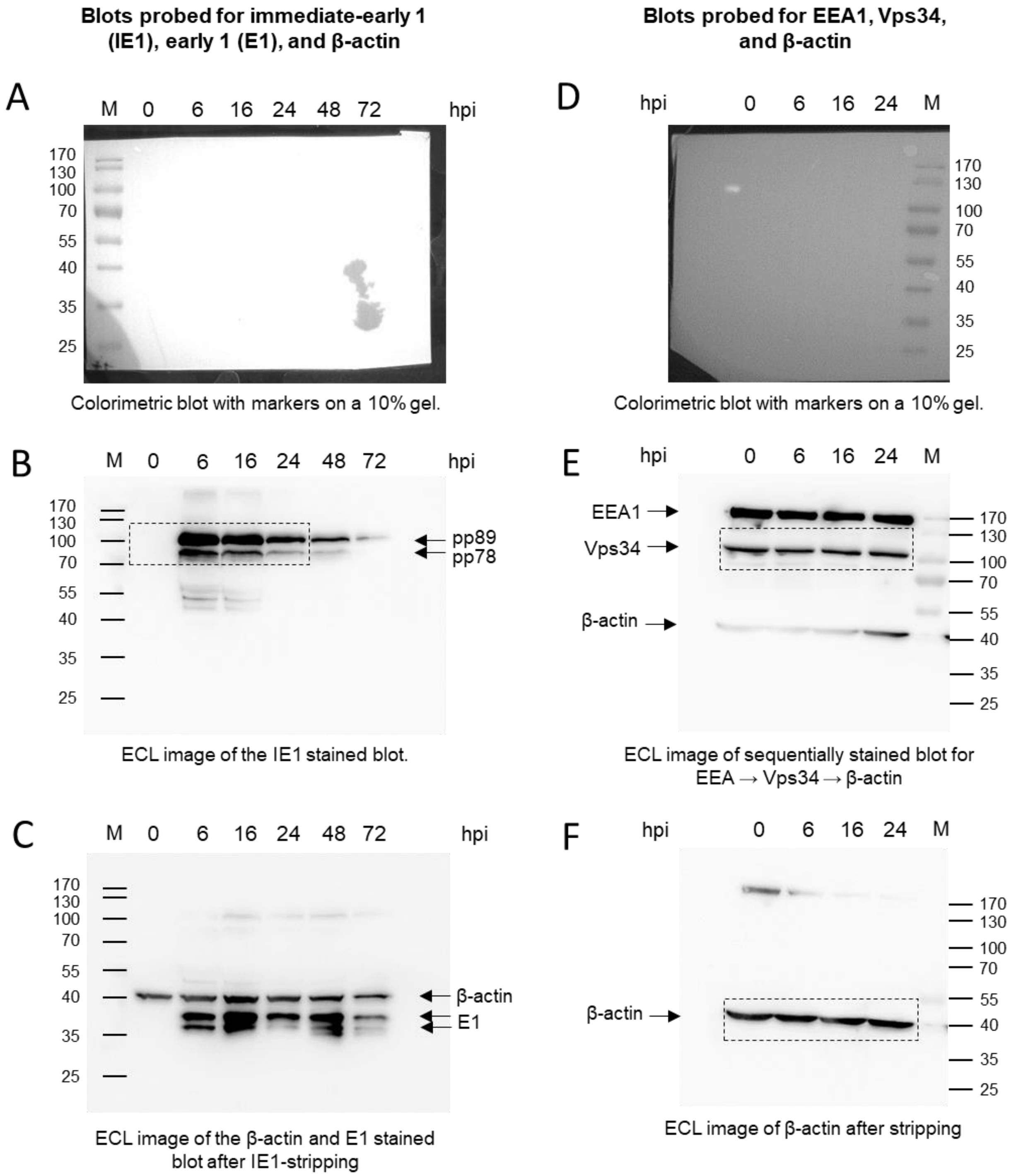

Figure S2. **Original raw blots and unedited ECL images of IE1, Vps34, and β-actin used as a representative Western blot in Figure 1C of the manuscript.** (**A**) Original raw blot used for staining of IE1 (**B**) and subsequent staining of E1 and β-actin (**C**) after stripping off the anti-IE1. The IE1 protein is displayed as a phosphoprotein of 89 kDa (pp89) and as pp78 form. The E1 protein is expressed in 38 and 36 forms. (**D**) Original raw blot used for staining EEA1, Vps34, and β-actin (**E**). The same blot was stripped and re-stained for β-actin (**F**). The expected molecular weight of EEA1 was 160 kDa, and Vps34 was 100 kDa. The molecular weight markers used in these experiments are PageRuler Prestained Protein Ladder (Thermo Scientific, Product: 26616, LOT: 00515508). For blotting, we used PVDF membrane (Merck Millipore, size:0,45um, Immobilon-P Transfer Membranes CAT: IPVH00010, LOT: R6PA1239C). The dashed-line area box indicates cropped area used for the assembly of Figure 1C. M, marker line; 0-72, preparation time of infected-cell samples; hpi, hours post-infection.

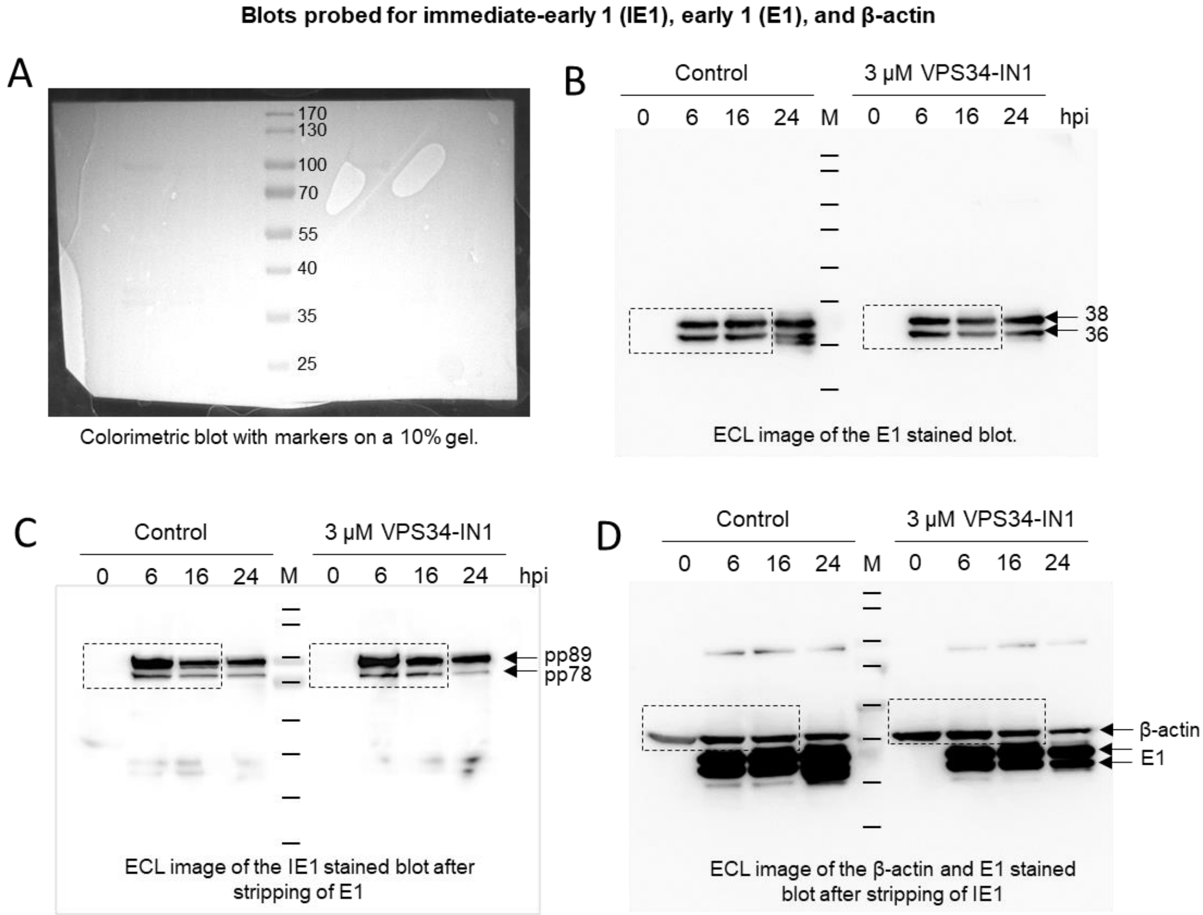

Figure S3. **Original raw blots and unedited ECL images of IE1, E1, and β-actin used as a representative Western blot in Figure 2C of the manuscript. (A)** Original raw blot used for staining of E1 **(B),** subsequent staining of IE1 after stripping (**C**), and staining of β-actin after stripping **(D).** The IE1 protein is displayed as a phosphoprotein of 89 kDa (pp89) and as pp78 form. The E1 protein is expressed in 38 and 36 forms. The molecular weight markers used in these experiments are PageRuler Prestained Protein Ladder (Thermo Scientific, Product: 26616, LOT: 00515508). For blotting, we used PVDF membrane (Merck Millipore, size:0,45um, Immobilon-P Transfer Membranes CAT: IPVH00010, LOT: R6PA1239C). The dashed-line area box indicates cropped area used for the assembly of Figure 2C. M, marker line; 0-24, preparation time of infected-cell samples; hpi, hours post-infection.

Blots probed for immediate-early 1 (IE1), early 1 (E1), and β-actin

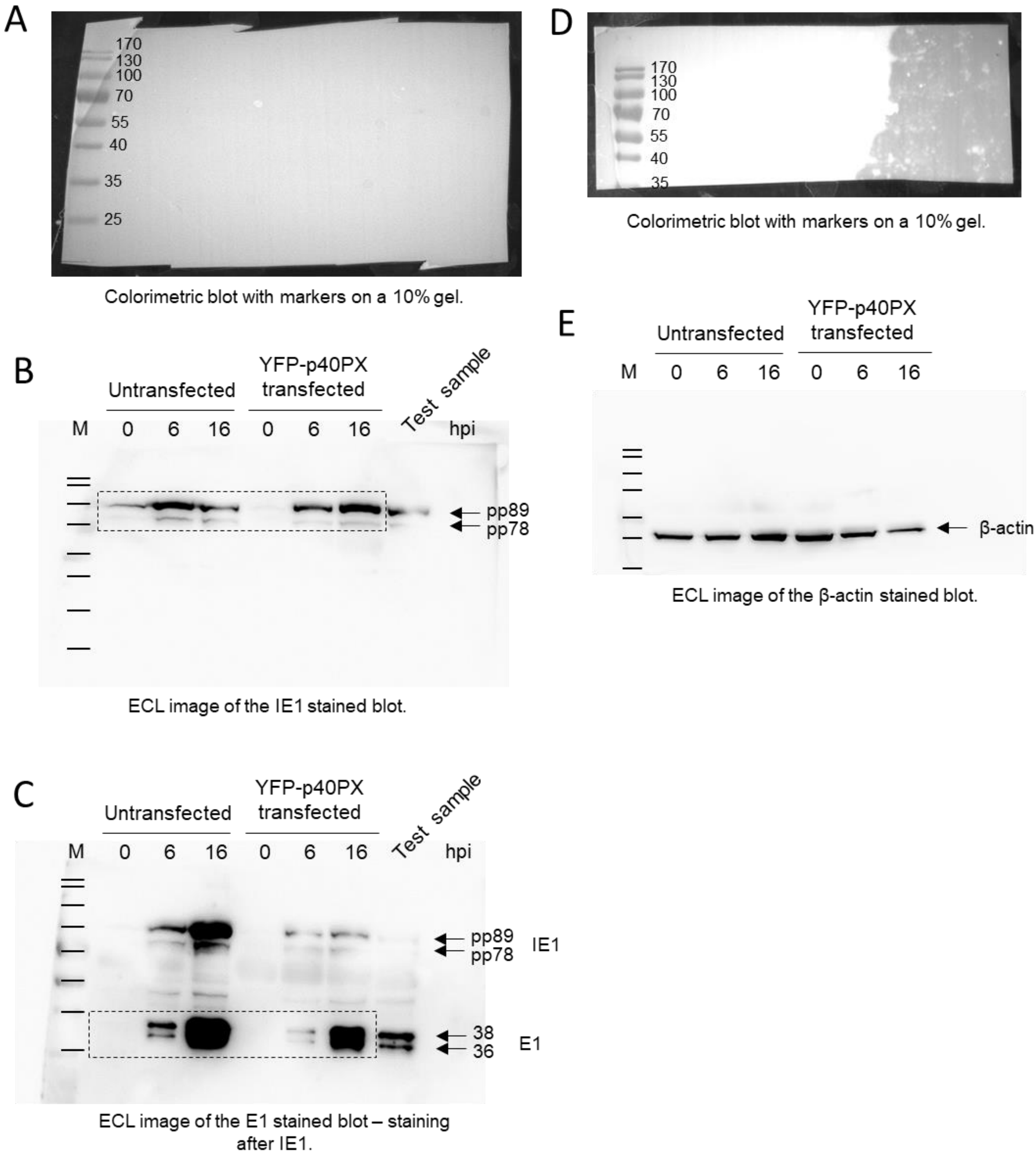

Figure S4. **Original raw blots and unedited ECL images of IE1, E1, and β-actin used as a representative Western blot in Figure 2D of the manuscript. (A)** Original raw blot used for staining of IE1 **(B),** subsequent staining of E1 (**C**). The IE1 protein is displayed as a phosphoprotein of 89 kDa (pp89) and as pp78 form. The E1 protein is displayed in 38 and 36 forms. (**D**) Original raw blot used for staining of β-actin **(E).** The molecular weight markers used in these experiments are PageRuler Prestained Protein Ladder (Thermo Scientific, Product: 26616, LOT: 00515508). For blotting, we used PVDF membrane (Merck Millipore, size:0,45um, Immobilon-P Transfer Membranes CAT: IPVH00010, LOT: R6PA1239C). The dashed-line area box indicates cropped area used for the assembly of Figure 2D. M, marker line; 0-16, preparation time of infected-cell samples; hpi, hours post-infection.
